# Non-malignant features of cancer predisposition syndromes manifesting in childhood and adolescence: a guide for the general pediatrician

**DOI:** 10.1007/s12519-024-00853-8

**Published:** 2024-12-06

**Authors:** Michaela Kuhlen, Andreas B. Weins, Nicole Stadler, Daniela Angelova-Toshkina, Michael C. Frühwald

**Affiliations:** 1 Pediatrics and Adolescent Medicine, Faculty of Medicine, University of Augsburg, 86156 Augsburg, Germany; 2 Augsburger Zentrum für Seltene Erkrankungen, Faculty of Medicine, University of Augsburg, 86156 Augsburg, Germany

**Keywords:** Cancer predisposition, Children, Non-malignant manifestations, Psychosocial needs, Surveillance

## Abstract

**Purpose:**

Cancer predisposition syndromes are genetic disorders that significantly raise the risk of developing malignancies. Although the malignant manifestations of cancer predisposition syndromes are well-studied, recognizing their non-malignant features is crucial for early diagnosis, especially in children and adolescents.

**Methods:**

A comprehensive literature search was conducted using the PubMed database, focusing on non-malignant manifestations of cancer predisposition syndromes in children and adolescents. Key sources included the Clinical Cancer Research pediatric oncology series and ORPHANET. Studies that described clinical signs and symptoms affecting specific organ systems were included.

**Results:**

Non-malignant dermatological features often serve as early indicators of cancer predisposition syndromes, including café-au-lait spots in Neurofibromatosis Type 1 and facial angiofibromas in Tuberous Sclerosis Complex. Neurological and developmental anomalies such as cerebellar ataxia in ataxia-telangiectasia and intellectual disabilities in neurofibromatosis type 1 and tuberous sclerosis complex are significant indicators. Growth and metabolic anomalies are also notable, including overgrowth in Beckwith–Wiedemann syndrome and growth hormone deficiency in neurofibromatosis Type 1. In addition, facial anomalies, ocular manifestations, hearing issues, and thyroid anomalies are prevalent across various cancer predisposition syndromes. For instance, hearing loss may be significant in neurofibromatosis Type 2, while thyroid nodules are common in *PTEN* hamartoma tumor syndrome and *DICER1* syndrome. Cardiovascular, abdominal, musculoskeletal, pulmonary, genitourinary manifestations, and prenatal deviations further complicate the clinical picture.

**Conclusions:**

Recognizing non-malignant features of cancer predisposition syndromes is essential for early diagnosis and management. This organ-specific overview furthers awareness among healthcare providers, facilitating timely genetic counseling, surveillance programs, and preventive measures, ultimately improving patient outcomes.

**Graphical abstract:**

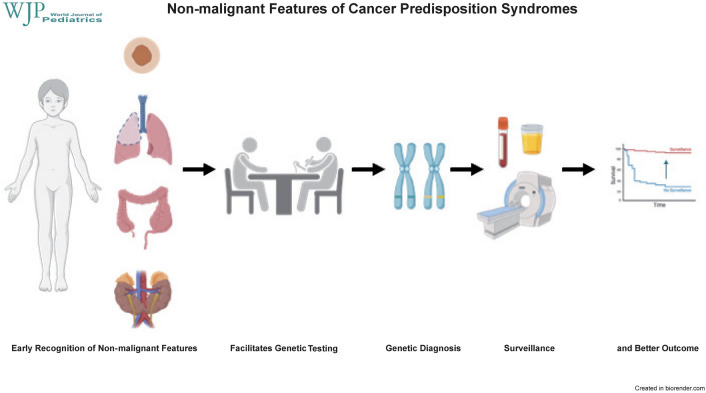

**Supplementary Information:**

The online version contains supplementary material available at 10.1007/s12519-024-00853-8.

## Introduction

Cancer predisposition syndromes (CPS) are a heterogeneous group of genetic disorders that significantly increase the lifetime risk of developing various malignancies [1–3]. These syndromes, often inherited in an autosomal dominant or recessive pattern, are characterized by germline variants among others in genes responsible for maintaining genomic stability, cell cycle control, DNA repair, and apoptosis [1, 4–6]. While malignant manifestations of CPS are well-documented and remain a primary focus of oncology research and clinical management [7–13], non-malignant signs and symptoms are crucial for early recognition and diagnosis [14–17].

Non-malignant manifestations encompass a wide range of clinical presentations affecting multiple organ systems, often preceding the development of malignancies or causing disabilities [14, 15, 17–19]. Pediatricians and other healthcare providers must maintain a high index of suspicion for CPS when encountering specific phenotypic anomalies, developmental delays, and other systemic manifestations in children and adolescents. Early identification of CPS through recognition of these non-malignant features facilitates timely genetic counseling, initiation of surveillance programs, and implementation of preventive measures, ultimately improving patient well-being and outcomes [1, 20].

**Fig. 1:**
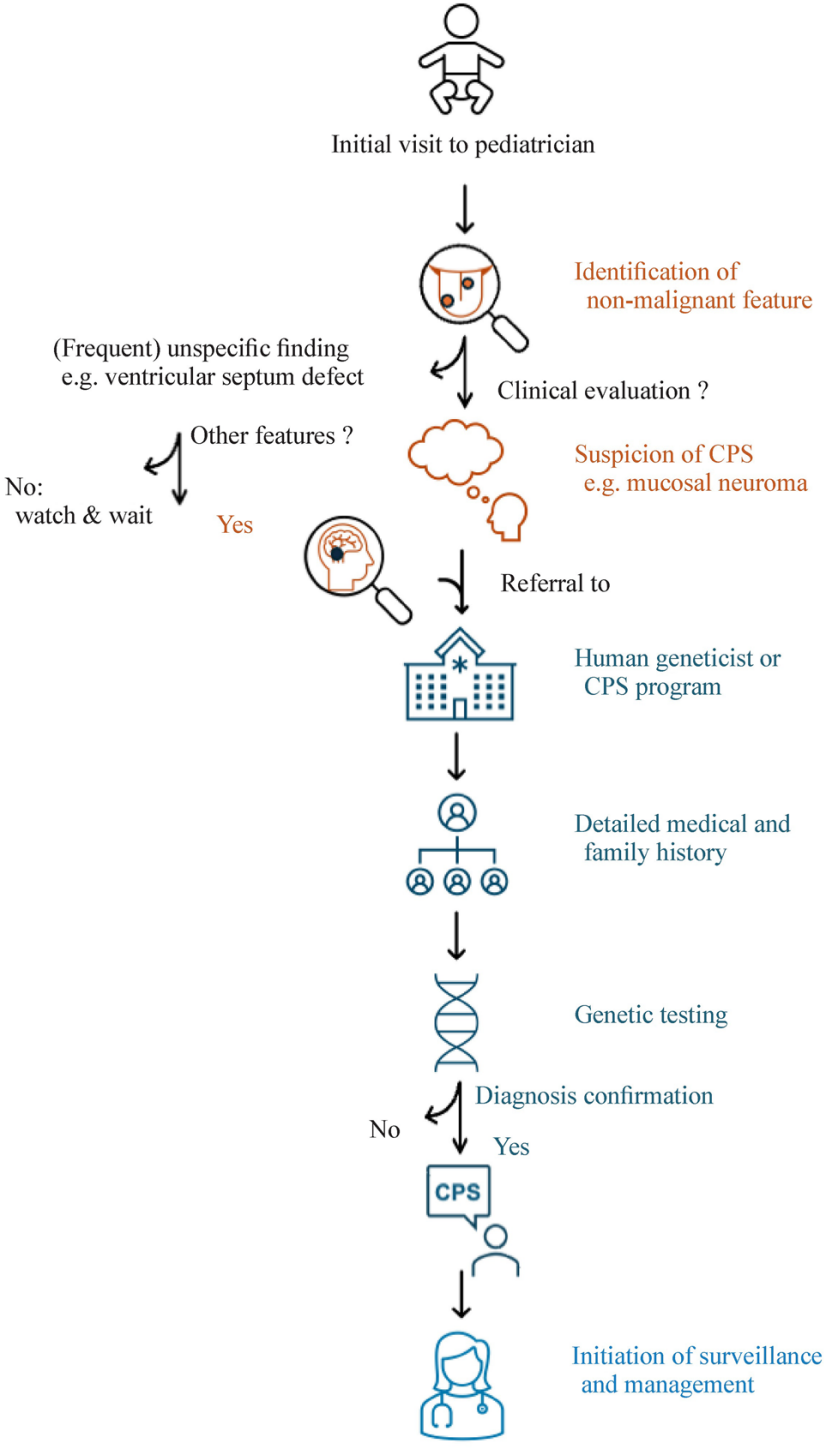
Flowchart of cancer predisposition syndrome (CPS) diagnosis process based on non-malignant features.This flowchart illustrates the step-by-step process for diagnosing CPS in pediatric patients, highlighting the role of recognizing non-malignant features, conducting genetic testing, and reaching a final diagnosis

In 2016, a workshop sponsored by the American Association for Cancer Research was held to develop consensus recommendations for cancer surveillance in children and adolescents with CPS. Experts including (co)directors of cancer predisposition programs (pediatric oncologists or medical geneticists), genetic counselors, radiologists, directors of adult cancer predisposition programs, and one pediatric endocrinologist were present. The resulting Clinical Cancer Research (CCR) pediatric oncology series provides a comprehensive overview and recommendations for surveillance of the 50 most common CPS, each carrying a 5% or greater cancer risk within the first 20 years of life [1, 20–33]. These recommendations were recently updated [34–36]. Building upon this fundamental work, the present review aims to provide an organ-specific overview of non-malignant signs and symptoms associated with CPS, offering a practical guide for daily practice (Fig. 1). This detailed exploration includes skin anomalies, neurological and developmental symptoms, growth and metabolic disorders, craniofacial dysmorphisms, ocular signs, head, neck, and thyroid anomalies, abdominal and gastrointestinal manifestations, musculoskeletal anomalies, pulmonary manifestations, cardiovascular signs, genitourinary issues, tumor development in a non-cancerous context, and prenatal deviations.

By augmenting awareness of these diverse clinical presentations, this review seeks to guide pediatricians and other healthcare providers in the early detection and multidisciplinary management of children and adolescents at risk for CPS.

## Methods

### Literature search

We conducted a comprehensive literature search to identify relevant publications on CPS and their respective non-malignant manifestations in children and adolescents. The primary sources of data were the CCR pediatric oncology series published in 2017, covering the 50 most common CPS with ≥ 5% cancer risk (Supplemental Table 1), as well as ORPHANET and GeneReviews ® [Internet] for information on non-malignant features of these CPS. Additionally, the PubMed database was searched using combinations of the following keywords: "cancer predisposition syndromes", "non-malignant symptoms", "non-neoplastic symptoms", "phenotypic manifestations", and specific syndrome names [e.g., neurofibromatosis type 1 (NF1), Beckwith–Wiedemann syndrome (BWS)]. CPS without non-malignant manifestations, e.g. Li-Fraumeni syndrome, were excluded from this analysis.

**Table 1:** Dermatological manifestations and associated cancer predisposition syndromes

Dermatological manifestations	Cancer predisposition syndrome
Acrochordons	Birt-Hogg-Dubé syndrome
Adenomas, sebaceous	Muir-Torre syndrome
Adipose tissue, subcutaneous sparse	Bloom syndrome
Alopecia	Dyskeratosis congenita
Angiofibromas, facial	Tuberous sclerosis complex
Ankle ulceration	Werner syndrome
Atrophy, (sub)cutaneous	Rothmund-Thomson syndrome, Werner syndrome
Café-au-lait macules	Bloom syndrome, cardiofaciocutaneous syndrome, constitutional mismatch repair deficiency, Fanconi anemia, NF1, Nijmegen-Breakage syndrome
Eczematous lesions	Cardiofaciocutaneous syndrome, Shwachman-Diamond syndrome
Epitheliomas, sebaceous	Muir-Torre syndrome
Erythema (cheeks, extremities, buttocks)	Rothmund-Thomson syndrome
Erythema, telangiectatic	Bloom syndrome
Fibrofolliculomas	Birt-Hogg-Dubé syndrome
Fibromas, ungual	Tuberous sclerosis complex
Freckling	Constitutional mismatch repair deficiency, NF1, xeroderma pigmentosum
Granulomas, cutaneous	Ataxia-Telangiectasia
Hair, curly	Cardiofaciocutaneous syndrome, Costello syndrome, Noonan syndrome,
Hamartomas, mucocutaneous	PTEN hamartoma tumor syndrome
Hemangioma	Cardiofaciocutaneous syndrome
Hyperkeratosis	Noonan syndrome, Rothmund-Thomson syndrome, Werner syndrome
Hyperpigmentation	Peutz-Jeghers syndrome, Rothmund-Thomson syndrome
Hypertrichosis	Schinzel-Giedion syndrome
Hypopigmentation	Rothmund-Thomson syndrome
Ichthyosis	Cardiofaciocutaneous syndrome, Shwachman-Diamond syndrome
Keloid formation	Rubinstein-Taybi syndrome
Keratoacanthomas	Muir-Torre syndrome
Keratoderma	Cardiofaciocutaneous syndrome
Leiomyomatosis, cutaenous	Hereditary leiomyomatosis and renal cell cancer
Lentiginosis, penis/vulva	Bannayan-Riley-Ruvalcaba syndrome
Leukoplakia, oral	Dyskeratosis congenita
Lichen amyloidosis, cutaneous	MEN2A
Lipomatosis, subcutaneous	Bannayan-Riley-Ruvalcaba syndrome
Lymphedema	Cardiofaciocutaneous syndrome, Noonan syndrome
Macules, dark blue to brown (mouth, eyes, nares, perianal, mucosal)	Peutz-Jeghers syndrome
Macules, hypomelanotic	Tuberous sclerosis complex
Nail, dystrophy	Rothmund-Thomson syndrome
Nails, dysplastic	Dyskeratosis congenita
Nail, hypoplasia	Simpson-Golabi-Behmel syndrome
Nails, hypoplastic/hyperconvex	Schinzel-Giedion syndrome
Neurofibromas	Constitutional mismatch repair deficiency, NF1
Neuromas, mucosal (lips, tongue)	MEN2B
Nevus flammeus	Beckwith-wiedemann syndrome, bohring-opitz syndrome, mulibrey nanism
Nevi	Cardiofaciocutaneous syndrome
Nevi, pigmented	Nijmegen-Breakage syndrome
Nipples, supernumerary	Simpson-Golabi-Behmel syndrome
Nodular tumors, subcutaneous	NF2
Palmar creases, single	Schinzel-Giedion syndrome
Papillomata	Costello syndrome
Pigmentation, reticular	Dyskeratosis congenita
Pits, palmar/plantar	Gorlin syndrome
Plaque-like lesions	NF2
Plaques, fibrous	Tuberous sclerosis complex
Skin, dry	Noonan syndrome, xeroderma pigmentosum
Skin, hyperkeratotic/hyperelastic	Cardiofaciocutaneous syndrome
Skin, loose/soft	Costello syndrome, Weaver syndrome
Skin lesions, “confetti”	Tuberous sclerosis complex
Skin lesion, hyperpigmented	Xeroderma pigmentosum
Skin lesions, hypopigmented	Bloom syndrome, xeroderma pigmentosum
Skin, tight	Werner syndrome
Telangiectasias	Ataxia-telangiectasia, Rothmund-Thomson syndrome
Trichodiscomas	Birt-Hogg-Dubé syndrome
Vascular malformations	Bannayan-Riley-Ruvalcaba syndrome, Beckwith-Wiedemann syndrome
Vitiligo spots	Nijmegen-Breakage syndrome

NF1 neurofibromatosis type 1, MEN2A Multiple Endocrine Neoplasia Type 2A, NF2 neurofibromatosis type 2

### Inclusion and exclusion criteria

Studies were included whenever they met the following criteria:

1. Published in English.
2. Focused on non-malignant manifestations of CPS in childhood and adolescence.
3. Provided detailed descriptions of clinical signs and symptoms affecting specific organ systems.

Studies were excluded if they:

1. Focused solely on adult populations.
2. Did not differentiate between malignant and non-malignant symptoms.
3. Focused on CPS without non-malignant manifestations, e.g. Li-Fraumeni syndrome.

### Data extraction and synthesis

**Fig. 2:**
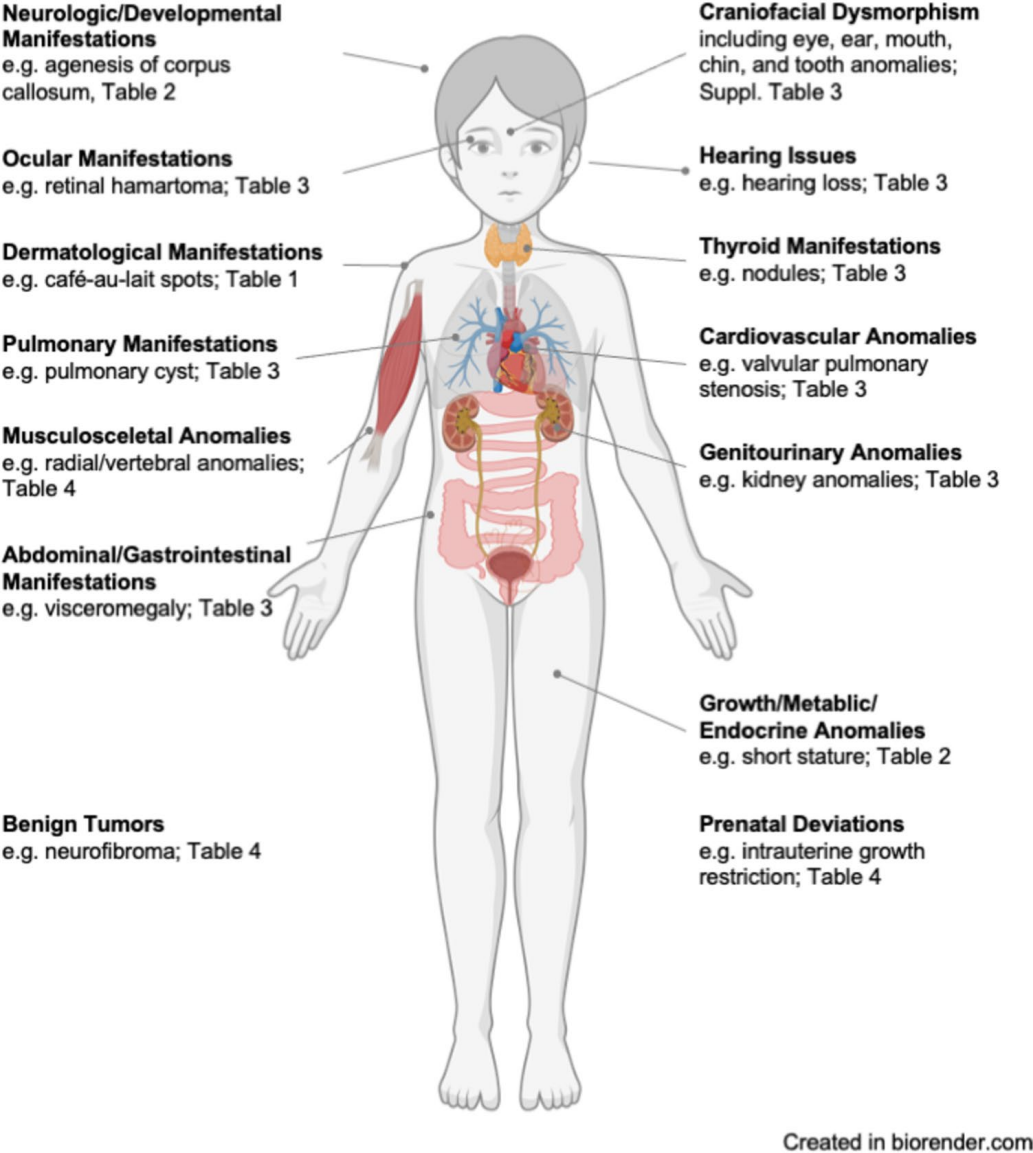
Organ-specific manifestations. Infographic showing which organ systems are affected by different cancer predisposition syndromes

Extracted data included syndrome names, affected organ systems, specific non-malignant signs and symptoms, frequency of signs and symptoms, and age at first occurrence, if applicable (Supplemental Table 2). No further exploration of hematological manifestations was performed. A structured approach was used to categorize the findings by organ system (Fig. 2), ensuring a comprehensive overview relevant to daily clinical practice.

**Table 2:** Neurological, developmental, growth, metabolic, and endocrinological manifestations and associated cancer predisposition syndromes

Manifestations	Cancer predisposition syndrome
Neurological/developmental	
Apraxia, oculomotoric	Ataxia-telangiectasia
Arachnoid cyst	Nijmegen-breakage syndrome
Arnold-Chiari malformation	Costello syndrome
Ataxia	Ataxia-telangiectasia, xeroderma pigmentosum
Attention deficit/hyperactivity disorder	Noonan syndrome, tuberous sclerosis complex
Autism spectrum disorder	Bannayan-Riley-Ruvalcaba syndrome, tuberous sclerosis complex
Balance disorders	Ataxia-telangiectasia
Behavioral disorders	Sotos syndrome, WAGR syndrome
Choreoathetosis	Ataxia-telangiectasia
Choroid plexus hemangioma	Perlman syndrome
Cognitive deficits/impairments	NF1, xeroderma pigmentosum
Coordination, poor	Weaver syndrome
Corpus callosum, agenesis	Perlman syndrome
Corpus callosum defect	Bohring-Opitz syndrome
Cortical dysplasia	Tuberous sclerosis complex
Developmental delay	Alagille syndrome, Bannayan-Riley-Ruvalcaba syndrome, cardiofaciocutaneous syndrome, CBL syndrome, Costello syndrome, dyskeratosis congenita, Schinzel-Giedion syndrome, Sotos syndrome
Drooling	Ataxia-telangiectasia
Dyspraxia	Noonan syndrome
Epilepsy/seizures	Bohring-Opitz syndrome, cardiofaciocutaneous syndrome, Costello syndrome, NF1, Schinzel-Giedion syndrome, Sotos syndrome, tuberous sclerosis complex, xeroderma pigmentosum
Hydrocephalus	Costello syndrome, NF1, Nijmegen-Breakage syndrome
Hypertonia/Spasticity	Weaver syndrome, xeroderma pigmentosum
Hypotonia	Schinzel-Giedion syndrome, Sotos syndrome, Weaver syndrome
Intellectual decline	Nijmegen-Breakage syndrome
Intellectual disability	Costello syndrome, Noonan syndrome, Shwachman-Diamond syndrome, Simpson-Golabi-Behmel syndrome, Seckel syndrome, Sotos syndrome, tuberous sclerosis complex, WAGR syndrome, Weaver syndrome
Learning difficulties	Cardiofaciocutaneous syndrome, NF1, Noonan syndrome
Motor development, delayed	Noonan syndrome, Simpson-Golabi-Behmel syndrome
Neuropsychological deficits	Tuberous sclerosis complex
Psychiatric disorders	Tuberous sclerosis complex
Psychomotor retardation	Shwachman-Diamond syndrome
Schizencephaly	Nijmegen-Breakage syndrome
Slurred speech	Ataxia-telangiectasia
Speech, delayed	Noonan syndrome, Simpson-Golabi-Behmel syndrome
Subependymal nodules	Tuberous sclerosis complex
Syringomyelia	Costello syndrome
Tethered spinal cord	Costello syndrome
Growth, metabolic and endocrinological	
ACTH, excessive production	MEN2A
Bone age, advanced	Sotos syndrome
Bone age, delayed	Shwachman-Diamond syndrome
Endocrinopathy	Fanconi anemia
Failure to thrive	Costello syndrome, Noonan syndrome, Shwachman-Diamond syndrome
Glucose intolerance/insulin resistance	Ataxia-telangiectasia, Mulibrey nanism
Growth delay/deficiency/retardation	Alagille syndrome, ataxia-telangiectasia, Bloom syndrome, juvenile polyposis syndrome, Nijmegen-Breakage syndrome, Noonan syndrome, Shwachman-Diamond syndrome
Growth, excessive	Sotos syndrome
Growth failure	Cardiofaciocutaneous syndrome
Growth hormone deficiency	Cardiofaciocutaneous syndrome
Hemihyperplasia	Beckwith-Wiedemann syndrome
Hyperparathyroidism	MEN1, MEN2A
Hypoglycemia	Beckwith-Wiedemann syndrome
Hypogonadism	Mulibrey nanism
Hyperinsulinism	Perlman syndrome
Ovarian insufficiency	Nijmegen-Breakage syndrome
Parathyroid hyperplasia/hypercalcemia	Hyperparathyroid-jaw tumor syndrome
Puberty, delayed/disordered	Costello syndrome, Frasier syndrome, Noonan syndrome
Short stature	Costello syndrome, Diamond-Blackfan anemia, dyskeratosis congenita, Fanconi anemia, NF1, Noonan syndrome, Shwachman-Diamond syndrome, Werner syndrome
Tall stature	Weaver Syndrome

NF1 neurofibromatosis type 1, WAGR Wilms tumor, aniridia, genitorurinary abnormalities, and range of developmental delays syndrome, MEN2A Multiple Endocrine Neoplasia Type 2A, ATCTH Adrenocorticotropic hormone

## Results

### Dermatological manifestations

**Fig. 3:**
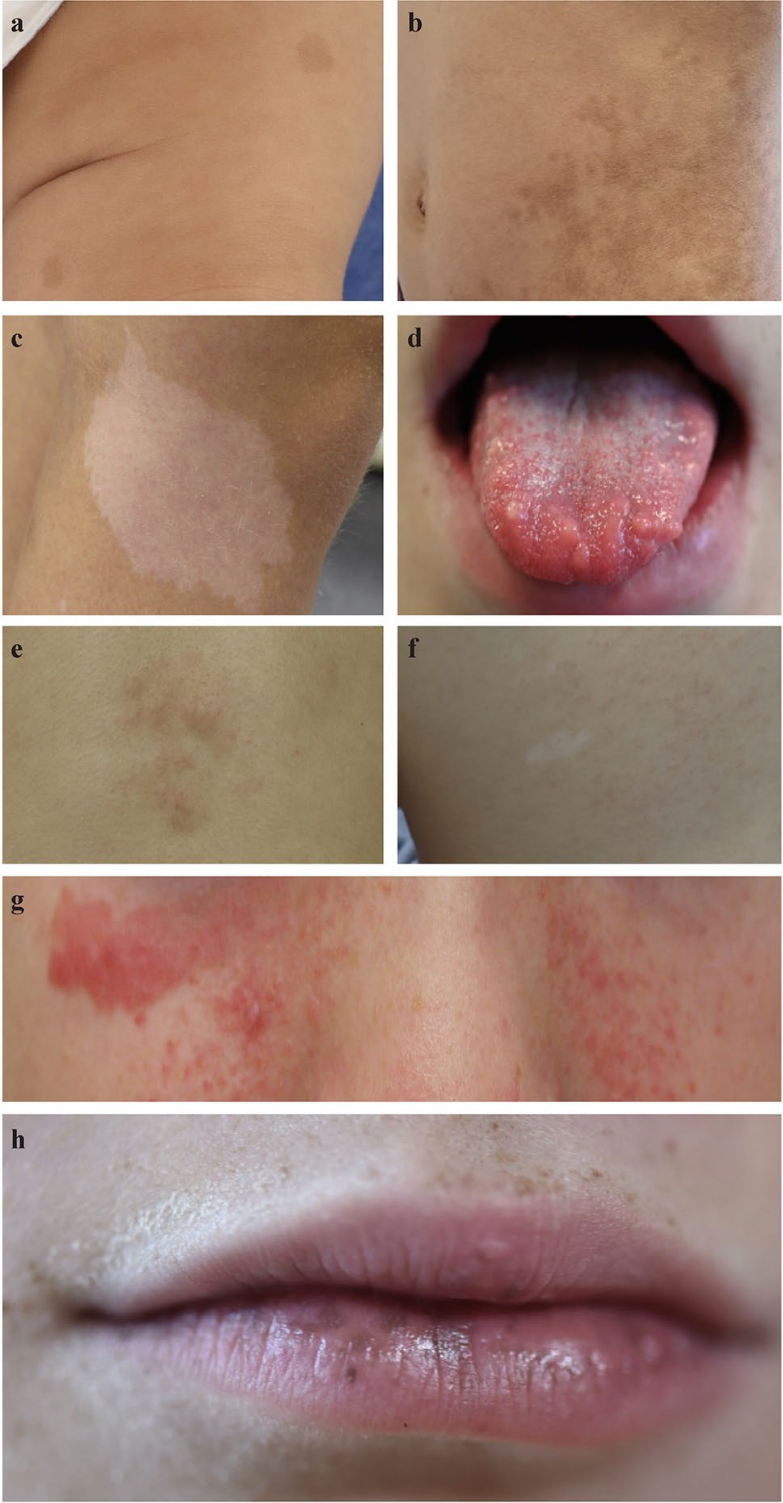
Illustrative photos of key dermatological signs: **a** typical café-au-lait spots; **b** atypical café-au-lait spots; **c** hypomelanotic macule; **d** mucosal neuromas; **e** Shagreen patches; **f** white spots; **g** facial angiofibromas; **h** (mucocutaneous) perioral pigmentation

Non-malignant dermatological features (Table 1) are often among the earliest and most apparent indicators of CPS. Café-au-lait spots, frequently associated with NF1 [37–39] and constitutional mismatch repair deficiency (CMMRD) [15, 40, 41], are light brown skin lesions that vary in size and number (Fig. 3a, b). Patients with NF1 typically have six or more spots, which are crucial for diagnosis [37, 39]. These spots often appear in early childhood and may be accompanied by axillary or inguinal freckling. Facial angiofibromas, common to tuberous sclerosis complex (TSC) [42–45], present as red or flesh-colored papules primarily on the nose and cheeks (Fig. 3e). Other notable dermatological features include hyperpigmentation and telangiectasias in Bloom syndrome (BS) [46, 47] due to increased sun sensitivity, sebaceous adenomas in Muir–Torre syndrome (MTS) [48, 49], palmar or plantar pits in Gorlin syndrome (GS) [50–53], and mucocutaneous perioral pigmentation in Peutz–Jeghers syndrome (PJS) [54–56] (Fig. 3f). Conditions such as xeroderma pigmentosum (XP) [57, 58] and Rothmund–Thomson syndrome [59, 60] are characterized by severe photosensitivity, poikiloderma, and cutaneous atrophy, necessitating strict photoprotection to prevent skin damage and malignancy. Additionally, Birt–Hogg–Dubé syndrome (BHDS) [61] often presents with fibrofolliculomas, trichodiscomas, and acrochordons. Rubinstein–Taybi syndrome (RTS) [62] features keloid formation, while Cowden syndrome, part of *PTEN* hamartoma tumor syndrome (PHTS) [54, 63, 64], features pigmentation anomalies such as trichilemmomas and papillomatous papules.

### Neurological and developmental features

Neurological and developmental anomalies (Table 2) are significant indicators of CPS. Cerebellar ataxia, a hallmark of ataxia-telangiectasia (A-T) [65–67], manifests as a progressive loss of coordination and balance. Patients exhibit oculomotor apraxia, making eye movements difficult. Intellectual and learning disabilities are prevalent in NF1 [37–39, 68], TSC [42–45], and PHTS [54, 63, 64], posing substantial challenges during cognitive development. Seizures and autism spectrum disorder are primarily associated with TSC [42–45], with seizures often being refractory and difficult to manage. Macrocephaly is a common feature in both NF1 [37–39, 68] and PHTS [54, 63, 64], and in the context of CPS, it often coexists with other dysmorphic features or neurological anomalies. Furthermore, developmental delay, hypotonia, and speech difficulties are common to several syndromes, such as Costello syndrome (CS) [69, 70] and cardiofaciocutaneous (CFC) syndrome [71]. Patients with BS [46, 47] may demonstrate normal intelligence but suffer from speech and drooling issues that can be misinterpreted as intellectual deficiency.

### Growth and metabolic anomalies

CPS can profoundly affect growth and metabolism (Table 2). BWS [72, 73] is characterized by both prenatal and postnatal overgrowth, as well as associated features such as macroglossia, which can lead to difficulties in feeding and speech. Hyperinsulinism, leading to hypoglycemia, is a common metabolic issue in BWS [72, 73]. BS [46, 47] often presents with failure to thrive due to growth difficulties, proportionate growth deficiency of prenatal onset, and continued growth deficiency throughout life. Growth hormone deficiency, often seen in NF1 [37–39] and Fanconi anemia (FA) [67, 74, 75], results in short stature and other related complications. Additionally, conditions such as Simpson–Golabi–Behmel syndrome [76–78] feature prenatal and postnatal overgrowth, macrocephaly, and macroglossia. Patients with CS [69, 70] exhibit severe postnatal feeding difficulties, failure to thrive, and short stature.

### Craniofacial dysmorphism

Facial anomalies (Supplemental Table 3) are common non-malignant indicators of many CPS and can serve as important diagnostic clues. Proteus syndrome (PS) [54, 79, 80] can result in asymmetric overgrowth of facial features, contributing to a distinctive facial appearance. In multiple endocrine neoplasia type 2B (MEN2B) [17, 81, 82], mucosal neuromas (Fig. 3d) cause bumpy, often enlarged lips and tongue.

**Table 3:** Organ-specific manifestations including ocular manifestations, hearing issues, dental issues, thyroid, pulmonary, cardiovascular, abdominal/gastrointestinal, and genitourinary manifestations and associated cancer predisposition syndromes

Manifestations	Cancer predisposition syndrome
Ocular manifestations	
Alacrima	MEN2B
Aniridia	WAGR syndrome
Axenfeld anomaly	Alagille syndrome
Blepharitis	Dyskeratosis congenita
Cataract	Gorlin syndrome, NF2, WAGR syndrome, Werner syndrome
Coloboma	Gorlin syndrome
Corneal nerve, prominent	MEN2B
Corneal opacification/vascularization	WAGR syndrome
Embryotoxon, posterior	Alagille syndrome
Epiphora	Dyskeratosis congenita
Glaucoma (congenital)	Rubinstein-Taybi syndrome, WAGR syndrome
Iris hamartomas	NF1
Keratitis	Xeroderma pigmentosum
Microphthalmus	Fanconi anemia, Gorlin syndrome
Myopia	Bohring-Opitz syndrome
Nasolacrimal duct obstruction	Rubinstein-Taybi syndrome
Nystagmus	Cardiofaciocutaneous syndrome
Optic nerve hypoplasia	WAGR syndrome
Photophobia	Xeroderma pigmentosum
Retinal hamartoma	Tuberous sclerosis complex
Retinal hemangioblastoma	Von-Hippel-Lindau syndrome
Retinal/optic nerve anomalies	Bohring-Opitz syndrome, cardiofaciocutaneous syndrome
Retinal mid-peripheral region, yellowish dots	Mulibrey nanism
Retinal pigment epithelium, hypertrophy	Familial adenomatous polyposis
Retinopathy, pigmentary	Alagille syndrome
Papillary and optic disc anomalies	Alagille syndrome
Strabismus	Cardiofaciocutaneous syndrome, Noonan syndrome
Telangiectasias, retinal	Ataxia-telangiectasia
Visual impairment	Schinzel-Giedion syndrome
Hearing issues	
Deafness	NF2
Endolymphatic sac tumor	Von-Hippel-Lindau syndrome
Hearing loss	Fanconi anemia, NF2, Noonan syndrome, Sotos syndrome, xeroderma pigmentosum
Hearing impairment	Schinzel-Giedion syndrome
Dental issues	
Dental anomalies	Rothmund-Thomson syndrome, Shwachman-Diamond syndrome
Dental crowding	Noonan syndrome
Dental enamel pitting	Tuberous sclerosis complex
Dental eruption, premature	Sotos syndrome
Dental malocclusion	Simpson-Golabi-Behmel syndrome
Dentigerous cysts	Familial adenomatous polyposis
Jaw fibromas, ossifying	Hyperparathyroid-jaw tumor syndrome
Keratocysts, mandibular odontogenic	Gorlin syndrome
Periodontal disease	Dyskeratosis congenita
Taurodontism	Dyskeratosis congenita
Teeth/root ratio, decreased	Dyskeratosis congenita
Teeth, supernumerary	Familial adenomatous polyposis
Teeth, unerupted	Familial adenomatous polyposis
Thyroid manifestations	
Hashimoto thyroiditis	Bannayan-Riley-Ruvalcaba syndrome, *PTEN* hamartoma tumor syndrome
Hypothyroidism	Alagille syndrome
Thyroid dysfunction	Noonan syndrome
Thyroid dysplasia	*DICER1* syndrome
Thyroid nodules	*DICER1* syndrome, *PTEN* hamartoma tumor syndrome
Pulmonary manifestations	
Airway infections, recurrent	Ataxia-telangiectasia, Nijmegen-Breakage syndrome, Rubinstein-Taybi syndrome
Bronchiectasis	Ataxia-telangiectasia
Chylothorax	Tuberous sclerosis complex
Cysts, (sub)pleural	Birt-Hogg-Dubé syndrome
Diaphragmatic hernia	Perlman syndrome, Simpson-Golabi-Behmel syndrome
Lymphangioleiomyomatosis	Tuberous sclerosis complex
Pneumocyte hyperplasia, multifocal micronodular	Tuberous sclerosis complex
Pneumothorax	Birt-Hogg-Dubé syndrome, tuberous sclerosis complex
Pulmonary arteriovenous malformations	Dyskeratosis congenita
Pulmonary cyst	*DICER1* syndrome, tuberous sclerosis complex
Pulmonary fibrosis	Dyskeratosis congenita
Sleep apnea, Obstructive	Bohring-Opitz syndrome
Cardiovascular anomalies	
Aortic arch, interrupted	Perlman syndrome
Arrhythmia	Costello syndrome, Simpson-Golabi-Behmel syndrome
Cardiac malformations	Beckwith-Wiedemann syndrome, Simpson-Golabi-Behmel syndrome, Sotos syndrome
Cardiomyopathy, hypertrophic	Cardiofaciocutaneous syndrome,
Coronary arteries, dilatation	Noonan syndrome
Dextroposition, heart	Perlman syndrome
Ductus arteriosus, patent	Alagille syndrome, Rubinstein-Taybi syndrome, Schinzel-Giedion syndrome
Hypertension	NF1
Moyamoya disease	NF1, Noonan syndrome
Perimyocardial heart disease, restrictive	Mulibrey nanism
Pulmonary artery stenosis or atresia	Alagille syndrome
Septal defects, atrial and/or ventricular	Alagille syndrome, Bohring-Opitz syndrome, Noonan syndrome, Rubinstein-Taybi syndrome, Schinzel-Giedion syndrome
Tetralogy of Fallot	Alagille syndrome
Valvular dysplasia	Schinzel-Giedion syndrome
Valvular pulmonary stenosis	Cardiofaciocutaneous syndrome, Costello syndrome, Noonan syndrome
Vascular malformation	*PTEN* hamartoma tumor syndrome
Ventricles, hypoplastic	Schinzel-Giedion syndrome
Abdominal/gastrointestinal manifestations	
Anorectal malformations	Fanconi anemia
Cholestasis, chronic	Alagille syndrome
Constipation, chronic	Cardiofaciocutaneous syndrome, MEN2B, Rubinstein-Taybi syndrome
Diarrhea	MEN2A, MEN2B
Diastasis recti	Beckwith-Wiedemann syndrome
Duodenal atresia	Fanconi anemia
Esophageal atresia	Fanconi anemia
Esophageal stenosis	Dyskeratosis congenita
Ganglioneuromatosis, generalized	MEN2B
Hepatic fibrosis	Perlman syndrome
Hepatomegaly	Mulibrey nanism
Hernia, umbilical	Beckwith-Wiedemann syndrome, Simpson-Golabi-Behmel syndrome, Weaver syndrome
Hirschsprung disease	MEN2A
Ileal atresia, distal	Perlman syndrome
Liver disease	Dyskeratosis congenita
Liver, fatty	Mulibrey nanism
Megacolon	MEN2B
Pancreatic cysts	Von-Hippel-Lindau syndrome
Pancreatic insufficiency, exocrine	Shwachman-Diamond syndrome
Steatohepatitis	Ataxia-telangiectasia
Telangiectasias, gastrointestinal	Dyskeratosis congenita
Visceromegaly	Beckwith-Wiedemann syndrome, Perlman syndrome, Simpson-Golabi-Behmel syndrome
Genitourinary anomalies	
Anus, anteriorly displaced	Schinzel-Giedion syndrome
Azo-/oligospermia	Bloom syndrome
Cryptorchidism	Nijmegen-Breakage syndrome, Noonan syndrome, Perlman syndrome, Schinzel-Giedion syndrome, Simpson-Golabi-Behmel syndrome
Epididymal cysts/cystadenomas	Von-Hippel-Lindau syndrome
Genitalia, ambiguous external	Denys-Drash syndrome
Gonadal dysgenesis	Frasier syndrome
Hydronephrosis	Schinzel-Giedion syndrome
Hypoplastic labia majora/minora	Schinzel-Giedion syndrome
Hypoplastic uterus	Schinzel-Giedion syndrome
Hypospadias	Nijmegen-Breakage syndrome, Schinzel-Giedion syndrome, Simpson-Golabi-Behmel syndrome
Impaired fertility/infertility	Ataxia-telangiectasia, Fanconi anemia, Frasier syndrome, Mulibrey nanism, Noonan syndrome
Labial sulcus, deep	Schinzel-Giedion syndrome
Kidney, ectopic/dystopic	Nijmegen-Breakage syndrome
Kidney, Horseshoe	Nijmegen-Breakage syndrome
Kidneys, small/dysplastic	Alagille syndrome, Simpson-Golabi-Behmel syndrome
Kidney-related anomalies	Beckwith-Wiedemann syndrome, Denys-Drash syndrome
Micropenis	Schinzel-Giedion syndrome
Nephroma, cystic	*DICER1* syndrome
Nephropathy	Frasier syndrome
Nephrotic syndrome	Denys-Drash syndrome, Frasier syndrome
Ovary hypoplasia	Nijmegen-Breakage syndrome
Renal cysts	Hyperparathyroid-jaw tumor syndrome, Von-Hippel-Lindau syndrome
Renal disease, end-stage	Frasier syndrome, Hyperparathyroid-jaw tumor syndrome, WAGR syndrome
Testicular development, disorder	Denys-Drash syndrome
Urethral stenosis	Dyskeratosis congenita
Urogenital anomalies	Diamond-Blackfan anemia, Sotos syndrome, WAGR Syndrome
Urolithiasis	Hyperparathyroid-jaw tumor syndrome

NF1 neurofibromatosis type 1, NF2 neurofibromatosis type 2, MEN2A Multiple Endocrine Neoplasia Type 2A, MEN2B Multiple Endocrine Neoplasia Type 2B

A broad or prominent forehead is observed in several CPS. For instance, patients with Noonan syndrome (NS) [83, 84] typically present with a broad forehead, while those with CFC syndrome [71] exhibit a large forehead. Bohring–Opitz syndrome (BOS) [85] also includes a prominent forehead with glabellar nevus flammeus. RTS [62] is characterized by highly arched eyebrows and long eyelashes. Patients with NS [83, 84] may present with hypertelorism and ptosis, as well as downslanting palpebral fissures. In CS [69, 70], ptosis and full cheeks are common, while BOS [85] features synophrys, proptosis, and hypertelorism. A convex nasal ridge is typical in RTS [62], whereas patients with CFC syndrome [71] often present with a short nose with a depressed nasal bridge. BOS [85] can feature a depressed, wide nasal bridge and anteverted nares. NS [83, 84] patients frequently have a short nose with a depressed nasal bridge as well. Patients with CS [69, 70] often have a large mouth with prominent lips and thickened ear helices. Ear anomalies are also notable in several CPS; low-set, posteriorly rotated ears are common in NS [83, 84], while patients with CFC syndrome [71] may exhibit low-set ears as well.

### Ocular manifestations

Non-malignant ocular symptoms (Table 3) include retinal hamartomas in TSC [42–45] and Lisch nodules, which are pigmented iris hamartomas, in NF1 [37–39]. Patients with Wilms tumor-aniridia-genitourinary anomalies-intellectual disability (WAGR) syndrome [86, 87] frequently exhibit aniridia, which can be associated with other eye anomalies such as cataract, glaucoma, and optic nerve hypoplasia. Additionally, conditions like BOS [85] can present with retinal and optic nerve anomalies, high myopia, and other vision issues.

### Hearing issues

Hearing loss (Table 3), both sensorineural and conductive, is associated with neurofibromatosis type 2 (NF2) [88–90]. Patients with NF2 often develop bilateral vestibular schwannomas, leading to progressive hearing loss.

### Thyroid manifestations

Thyroid anomalies (Table 3) are prevalent in several CPS. For example, PHTS [54, 63, 64] often presents with thyroid nodules and a predisposition to thyroid cancer. *DICER1* syndrome [64, 91, 92] is associated with multinodular goiter and differentiated thyroid cancer.

### Pulmonary manifestations

Pulmonary cysts leading to spontaneous pneumothorax are characteristic of BHDS [61]. In *DICER1* syndrome [64, 91, 92], cystic lesions in terms of pleuropulmonary blastoma (PPB) type I may precede the more aggressive malignant types II and III. Chronic respiratory infections due to immunodeficiency are common in A-T [65–67], and pulmonary fibrosis is a significant concern in dyskeratosis congenita [93, 94]. Patients with TSC [42–45] may develop lymphangioleiomyomatosis and multifocal micronodular pneumocyte hyperplasia.

### Cardiovascular anomalies

Congenital heart defects such as pulmonary valve stenosis and hypertrophic cardiomyopathy are associated with NS [83, 84] and CS [69, 70]. Patients with CFC syndrome [71] often present with valvular pulmonary stenosis and hypertrophic cardiomyopathy. Vascular anomalies, including arterial stenosis and aneurysms, are notable in NF1 [37–39, 68] and BS [46, 47], whereas cardiac fibromas are typical in GS [50–53]. Vascular malformations are also significant, with conditions like Bannayan–Riley–Ruvalcaba syndrome [54, 63, 95] presenting with hemangiomas and vascular anomalies, and PS [54, 79, 80] featuring vascular malformations, which may be capillary, venous, or lymphatic in nature.

### Abdominal and gastrointestinal manifestations

Hepatomegaly and nephromegaly are frequent findings in BWS [72, 73], while splenomegaly is common in FA [67, 74, 75]. Additionally, gastrointestinal anomalies such as esophageal and duodenal atresia may occur in individuals with FA, contributing to feeding difficulties and requiring surgical interventions early in life. Cystic nephromas, which are benign kidney tumors, are often seen in *DICER1* syndrome [64, 91, 92]. PJS [54–56] presents with gastrointestinal polyps, which may be detected at any site within the GI tract, most frequently in the small intestine, and may lead to complications such as intussusception.

### Genitourinary anomalies

Renal cysts and tumors are prevalent in Von Hippel-Lindau (VHL) disease [96–98] and BHDS [61]. Ambiguous genitalia and disorders of sex development are indicative of WAGR syndrome [86, 87]. Denys–Drash syndrome [99] features nephropathy that progresses to end-stage renal disease, along with genital anomalies such as ambiguous genitalia.

### Musculoskeletal anomalies

Musculoskeletal anomalies (Table 4) include scoliosis and bone dysplasia in NF1 [37–39], and jaw cysts along with bifid ribs in GS [50–53]. MEN2B [17, 81, 82] is characterized by marfanoid habitus, pes cavus, pectus excavatum, and joint hyperextensibility. RTS [62] features joint hypermobility and skeletal dysplasia.

**Table 4:** Musculoskeletal anomalies, benign tumors, and prenatal deviations and associated cancer predisposition syndromes

Manifestations	Cancer predisposition syndrome
Musculoskeletal anomalies	
Achilles tendons, tight	Costello syndrome
Bones, slender long with thick cortex, narrow medullary channel	Mulibrey nanism
Brachydactyly	Simpson-Golabi-Behmel syndrome
Brachymelia, mesomelic	Schinzel-Giedion syndrome
Camptodactylia	Weaver syndrome
Clinodactylia	Weaver syndrome
Contractures	Bohring-Opitz syndrome
Elbows, flexion	Bohring-Opitz syndrome
Elbow, radial head dislocation	Bohring-Opitz syndrome
Extremities, hypertonic	Bohring-Opitz syndrome
Femoral epiphysis, slipped capital	MEN2B
Fibrous dysplasia	Mulibrey nanism
Foot deformity	Schinzel-Giedion syndrome
Hip dislocation/dysplasia	Bohring-Opitz syndrome, Costello syndrome
Hypotonia	Bohring-Opitz syndrome, cardiofaciocutaneous syndrome, CBL syndrome
Joints, hyperextensible/hypermobile	MEN2B, Rubinstein-Taybi syndrome
Joint laxity	Costello syndrome, Weaver syndrome
Limbs, short	Schinzel-Giedion syndrome
Marfanoid habitus	MEN2B
Metaphyseal dysplasia	Shwachman-Diamond syndrome
Muscular hypoplasia, abdominal	Perlman syndrome
Myopathy	Bannayan-Riley-Ruvalcaba syndrome
Osteoporosis/osteopenia	Costello syndrome, dyskeratosis congenita, NF1, Shwachman-Diamond syndrome, Werner syndrome
Pectus carinatum	Shwachman-Diamond syndrome
Pectus excavatum	Bannayan-Riley-Ruvalcaba syndrome, MEN2B, Weaver syndrome
Pectus deformity	Gorlin syndrome
Pes cavus	MEN2B
Polydactyly	Gorlin syndrome, Simpson-Golabi-Behmel syndrome
Pseudoarthrosis	NF1
Radial/thumb anomalies	Diamond-Blackfan anemia, Fanconi anemia
Rib anomalies	Gorlin syndrome
Skeletal dysplasia	Tuberous sclerosis complex
Scoliosis	Costello syndrome, Gorlin syndrome, MEN2B, NF1, Noonan syndrome, Sotos syndrome, Weaver syndrome
Sella turcica, J-shaped	Mulibrey nanism
Shoulders, internal rotation	Bohring-Opitz syndrome
Sphenoid wing	NF1
Sprengel deformity	Gorlin syndrome
Sternal deformity	Noonan syndrome
Syndactyly	Gorlin syndrome, Simpson-Golabi-Behmel syndrome
Talipes equinovarus	Noonan syndrome
Thoracic cage, small	Mulibrey nanism
Ulnar deviation of wrist, fingers	Bohring-Opitz syndrome, Costello syndrome
Vertebral anomalies	Alagille syndrome, Gorlin syndrome, NF1, Simpson-Golabi-Behmel syndrome
Benign tumors	
Adenoma	Constitutional mismatch repair deficiency
Adenoma, parathyroid	Hyperparathyroid-jaw tumor syndrome
Angiomyolipomas, renal	Tuberous sclerosis complex
Desmoid tumor	Familial adenomatous polyposis
Fibroelastoma, heart	Gorlin syndrome
Fibroma, intraoral	Tuberous sclerosis complex
Fibroma, ovarian	Gorlin syndrome
Giant cell astrocytoma, subependymal	Tuberous sclerosis complex
Hamartoma	Hyperparathyroid-jaw tumor syndrome, tuberous sclerosis complex
Hemangioblastoma	Von-Hippel-Lindau syndrome
Leiomyoma, cutaneous/uterine	Hereditary leiomyomatosis and renal cell cancer
Melanoma, ocular	Xeroderma pigmentosum
Meningeoma	Gorlin syndrome, NF2
Neurofibromas, plexiform	NF1
Neuroendocrine tumors	MEN1, tuberous sclerosis complex, Von-Hippel-Lindau syndrome
Oncocytoma, renal	Birt-Hogg-Dubé syndrome
Optic pathway glioma	NF1
Osteoma	Familial adenomatous polyposis
Pituitary tumors	MEN1
Polyposis, gastrointestinal hamartomatous	Bannayan-Riley-Ruvalcaba syndrome
Polyp	Constitutional mismatch repair deficiency, familial adenomatous polyposis, Hyperparathyroid-Jaw tumor syndrome, juvenile polyposis syndrome, *PTEN* hamartoma tumor syndrome
Polyps, hamartomatous	Peutz-Jeghers syndrome
Rhabdomyomas, cardiac	Tuberous sclerosis complex
Schwannomas	NF2
Sex cord tumors with annular tubules	Peutz-Jeghers syndrome
Perinatal deviations	
Birth length, high	Weaver syndrome
Birth length, low	Fanconi anemia
Birth weight, high	Costello syndrome, Weaver syndrome
Birth weight, low	Fanconi anemia
Dwarfism, prenatal onset	Seckel syndrome
Fetal adrenocortical cytomegaly	Beckwith-Wiedemann syndrome
Growth, excessive, intrauterine	Sotos syndrome
Growth restriction, intrauterine	Bohring-Opitz syndrome, Mulibrey nanism
Hydrops	Costello syndrome
Omphalocele	Beckwith-Wiedemann syndrome
Polyhydramnios	Cardiofaciocutaneous syndrome

NF1 neurofibromatosis type 1, NF2 neurofibromatosis type 2, MEN1 Multiple Endocrine Neoplasia Type 1, MEN2B Multiple Endocrine Neoplasia Type 2B, CBL Casitas B-lineage lymphoma syndrome

### Benign tumors

Benign tumors (Table 4), including lipomas, hamartomas, and adenomas, are commonly observed in MEN syndromes and PJS [54–56]. MTS [48, 49] features sebaceous adenomas and epitheliomas, while PHTS [54, 63, 64] presents with mucocutaneous hamartomas and thyroid pathology.

### Prenatal deviations

Polyhydramnios and preterm birth represent obstetric complications associated with BWS [72, 73] and TSC [42–45]. BWS [72, 73] may be associated with abdominal wall defects.

## Discussion

CPS present a multifaceted clinical challenge due to their broad spectrum of non-malignant and malignant manifestations. These syndromes, which include conditions such as NF1, TSC, and BWS, among others, often require a high index of suspicion and an interdisciplinary approach to care.

### Complexity of non-malignant manifestations

The non-malignant features of CPS affect nearly every organ system, necessitating comprehensive and continued evaluation (Fig. 2). For instance, dermatological manifestations such as café-au-lait spots, facial angiofibromas, and sebaceous adenomas serve as critical early indicators that should prompt further genetic evaluation (Fig. 1) [16, 19, 37, 44, 45, 49, 100]. These features are not merely cosmetic concerns but are pivotal in the early diagnosis of CPS, facilitating timely intervention and management [68, 101].

Neurological and developmental symptoms, such as intellectual disabilities, learning challenges, and cerebellar ataxia, significantly impact the quality of life and developmental trajectory of affected individuals. For instance, cerebellar ataxia in A-T leads to progressive loss of coordination and balance, severely affecting daily functioning [65, 66]. Intellectual disabilities in NF1 and TSC result in substantial cognitive challenges, highlighting the need for continuous monitoring and specialized educational support [45, 68, 100].

Growth and metabolic anomalies, including prenatal and postnatal overgrowth seen in BWS and growth hormone deficiency in various CPS, require close collaboration between endocrinologists, geneticists, and nutritionists [73, 102]. Effective management of these conditions has the potential to prevent severe complications and improve overall outcomes. For instance, hyperinsulinism in BWS can lead to life-threatening hypoglycemia, highlighting the need for vigilant metabolic monitoring and management.

### Importance of interdisciplinary care

The multi-system involvement characteristic of CPS underscores the necessity for interdisciplinary care [37, 96, 103, 104]. This approach ensures comprehensive management of both the non-malignant and malignant aspects of these syndromes. Pediatricians, dermatologists, neurologists, endocrinologists, cardiologists, geneticists, and other specialists must collaborate closely to provide holistic care tailored to the individual needs of each patient.

For instance, the management of NF1 requires regular assessments to monitor neurofibroma, alongside neurological evaluations to address cognitive and developmental issues [37, 39, 68, 100]. Similarly, patients with A-T benefit from coordinated care involving immunologists for immunodeficiency management and infection prophylaxis, neurologists for monitoring neurological symptoms, and oncologists for ongoing tumor surveillance [65–67].

Moreover, genetic counseling plays a crucial role in the care of families affected by CPS [20]. It helps in understanding the hereditary nature of these syndromes, provides risk assessments for family members, and informs reproductive decisions. Genetic counselors help navigate families through the complexities of genetic testing and the implications of the results.

### Surveillance and preventive measures

Early identification and regular surveillance are essential in managing CPS [105–107]. These measures enable the early detection of complications and help prevent the progression of symptoms. For example, regular MRI scans are essential for detecting and monitoring brain tumors in patients with *SUFU*-associated GS, while ultrasound screenings are vital for identifying abdominal tumors in BWS [23, 51, 73].

Preventive measures, including strict photoprotection in conditions like BS and XP, are vital in reducing the risk of skin cancers [67]. Additionally, prophylactic interventions such as thyroidectomy in MEN2 to prevent medullary thyroid carcinoma, exemplify proactive management strategies necessary in CPS care [14, 33].

### Psychosocial support

The psychosocial impact of CPS on patients and their families is significant [108]. Chronic conditions and the potential for malignancies pose substantial emotional and psychological challenges [109, 110]. Providing comprehensive psychosocial support, including counseling and access to support groups, is crucial in helping patients and their families cope with the stress and uncertainties associated with CPS [110, 111].

### Transition aspects for children and adolescents with cancer predisposition syndromes

Transitioning from pediatric to adult care is a critical period for children and adolescents with CPS [112, 113]. This transition must be meticulously planned and managed to ensure continuity of care, adherence to surveillance protocols, and psychosocial support. Adolescents with CPS often face unique challenges due to the lifelong nature of their conditions, the complexity of their medical needs, and the potential for both malignant and non-malignant manifestations. Effective transition planning should start early, involving a multidisciplinary team that includes pediatricians, oncologists, and adult care providers. This ensures that all aspects of the patient’s health are considered and addressed. Education about the genetic nature of CPS, potential health risks, and the importance of continued surveillance empowers patients to understand their condition better, promoting self-management and adherence to medical recommendations. A well-structured transition program can significantly improve health outcomes and quality of life for adolescents with CPS, fostering a smooth and effective shift from pediatric to adult healthcare systems.

In conclusion, this comprehensive overview highlights the diverse range of non-malignant manifestations associated with CPS, emphasizing the critical importance of early detection and multidisciplinary management. By recognizing these features, pediatricians and other healthcare providers facilitate timely diagnosis, genetic counselling, and appropriate surveillance. This proactive approach ultimately improves medical outcomes and the well-being of affected children, adolescents, and those at risk of CPS.

## Supplementary Information

Below is the link to the electronic supplementary material.

Supplementary file1 (DOCX 117 kb)

## Data Availability

Not required.
