# Supplementary material for: Non-malignant features of cancer predisposition syndromes manifesting in childhood and adolescence: a guide for the general pediatrician
Source: World J Pediatr. 2024 Dec 6;21(2):131–48. doi: 10.1007/s12519-024-00853-8 (PMC11885337; doi:10.1007/s12519-024-00853-8)
Supplement: Supplementary file 1 — Supplementary file1 (DOCX 117 kb) [file 12519_2024_853_MOESM1_ESM.docx]

**Supplemental Table 1.** List of Cancer Predisposing Syndromes

1. Li-Fraumeni Syndrome
2. DICER1 Syndrome
3. PTEN Hamartoma Tumor Syndrome
4. Hereditary Retinoblastoma
5. Neurofibromatosis Type 1 (NF1)
6. Neurofibromatosis Type 2 (NF2)
7. Von Hippel-Lindau Syndrome
8. Multiple Endocrine Neoplasia Type 1 (MEN1)
9. Multiple Endocrine Neoplasia Type 2 (MEN2)
10. Beckwith-Wiedemann Syndrome
11. Wilms Tumor Syndromes
12. Rhabdoid Tumor Predisposition Syndrome
13. Gorlin Syndrome
14. Hereditary Pheochromocytoma and Paraganglioma Syndrome
15. FAP (Familial Adenomatous Polyposis)
16. Juvenile Polyposis Syndrome
17. Peutz-Jeghers Syndrome
18. Cowden Syndrome
19. Hereditary Nonpolyposis Colorectal Cancer (Lynch Syndrome)
20. Ataxia-Telangiectasia
21. Bloom Syndrome
22. Fanconi Anemia
23. Xeroderma Pigmentosum
24. Nijmegen Breakage Syndrome
25. Werner Syndrome
26. Rothmund-Thomson Syndrome
27. Schwachman-Diamond Syndrome
28. Li-Fraumeni-Like Syndrome
29. Noonan Syndrome
30. Costello Syndrome
31. Cardiofaciocutaneous Syndrome
32. Tuberous Sclerosis Complex
33. Birt-Hogg-Dubé Syndrome
34. Hereditary Diffuse Gastric Cancer
35. Medullary Thyroid Cancer Syndromes
36. Hyperparathyroid-Jaw Tumor Syndrome
37. Hereditary Melanoma Syndromes
38. Dyskeratosis Congenita
39. Seckel Syndrome
40. Sotos Syndrome
41. Weaver Syndrome
42. Simpson-Golabi-Behmel Syndrome
43. CMMRD (Constitutional Mismatch Repair Deficiency) Syndrome
44. Schinzel-Giedion Syndrome
45. Hippel-Lindau Syndrome
46. Alagille Syndrome
47. Muir-Torre Syndrome
48. Hereditary Leiomyomatosis and Renal Cell Cancer
49. Bannayan-Riley-Ruvalcaba Syndrome
50. Hereditary Breast and Ovarian Cancer Syndromes (BRCA1/2 related)

**Supplemental Table 2.** Most common CPS with 5% or greater cancer risk during the first 20 years of life and non-malignant manifestations. Manifestations were extracted from ORPHANET.

| **ALAGILLE SYNDROME** OMIM [118450](https://omim.org/entry/118450)  **Liver and Bile Ducts:**   - Chronic cholestasis due to paucity of intrahepatic bile ducts - Prolonged jaundice in newborns due to conjugated hyperbilirubinemia - Hepatosplenomegaly - Hypercholesterolemia and hypertriglyceridemia - Coagulopathy - Pruritus and xanthomas   **Cardiac Abnormalities:**   - Pulmonary artery stenosis or atresia - Atrial and/or ventricular septal defects - Tetralogy of Fallot - Patent ductus arteriosus   **Skeletal Anomalies:**   - Vertebrae segmentation anomalies, such as butterfly hemivertebrae (~50%) - Minor skeletal abnormalities including shortening of the radius, ulna, and phalanges   **Characteristic Facial Features:**   - Prominent forehead - Deep-set eyes - Upslanting palpebral fissures - Hypertelorism - Flat nasal root - Pointed chin   **Ophthalmologic Abnormalities:**   - Posterior embryotoxon (75% of cases) - Axenfeld anomaly - Pigmentary retinopathy - Papillary and optic disc anomalies   **Growth and Development:**   - Growth delay - Fat malabsorption, which may lead to rickets - Sometimes developmental delay   **Renal Abnormalities:**   - Small and dysplastic kidneys (common in Alagille syndrome type 2)   **Endocrine Abnormalities:**   - Hypothyroidism |
| --- |
| **ATAXIA-TELANGIECTASIA** OMIM [208900](https://omim.org/entry/208900)  **Neurological Symptoms:**   - Ataxia starting at 1-2 years of age - Balance disorders - Slurred speech - Drooling - Oculomotor apraxia - Choreoathetosis appearing around 9-10 years of age and worsening progressively - Normal intelligence, though speech and drooling issues may be misinterpreted as intellectual deficiency   **Cutaneous and Mucosal Symptoms:**   - Telangiectasias especially of the conjunctivae, appearing around 3-6 years of age or later   **Immunodeficiency:**   - Recurrent airway infections (otitis, sinusitis, bronchitis, pneumonia) - Risk of bronchiectasis - Autoimmune/inflammatory features such as granulomas, mostly of the skin but also other organs - Severity of immunodeficiency varies widely:   - Severe lymphopenia (including severe combined immune deficiency)   - Severe hypogammaglobulinemia   - No overt biological abnormality in some patients   **Additional Features:**   - Growth delay - Infertility - Glucose intolerance - Non-alcoholic steatohepatitis |
| **BANNAYAN-RILEY-RUVALCABA SYNDROME** OMIM [158350](https://omim.org/entry/158350)  **Head and Neck:**   - Macrocephaly   **Thyroid:**   - Hashimoto thyroiditis   **Skin and Subcutaneous Tissues:**   - Lipomatosis - Vascular malformations - Speckled lentiginosis of the penis or vulva   **Gastrointestinal:**   - Gastrointestinal hamartomatous polyposis   **Musculoskeletal:**   - Possible myopathic processes in proximal muscles (not clearly confirmed) - Pectus excavatum - Joint hyperextensibility (not clearly confirmed) - Scoliosis (not clearly confirmed)   **Additional Features:**   - High birth weight (not clearly confirmed) - Developmental delay - Risk of autism spectrum disorder (especially in *PTEN* carriers) |
| **BECKWITH-WIEDEMANN SYNDROME** OMIM [130650](https://omim.org/entry/130650)  **Growth:**   - Increased growth rate during the second half of pregnancy and the first few years of life - Adult height typically within the normal range - Hemihyperplasia   **Oral and Facial Features:**   - Macroglossia leading to difficulties in feeding, speech, and occasionally sleep apnea - Recognizable facial gestalt that often normalizes by adulthood   **Metabolic:**   - Hypoglycemia in 30-50% of neonates   **Abdominal Wall Defects:**   - Omphalocele - Umbilical hernia - Diastasis recti   **Ear Anomalies:**   - Anterior earlobe crease(s) - Posterior helical pit(s)   **Skin and Vascular:**   - Nevus flammeus or other vascular malformations   **Organ Enlargement:**   - Visceromegaly   **Adrenal Abnormalities:**   - Fetal adrenocortical cytomegaly (considered pathognomonic)   **Renal Abnormalities:**   - Various kidney-related anomalies   **Rare Findings:**   - Cleft palate (rare)   **Cardiac Abnormalities:**   - Cardiac malformations in 9-34% of cases - Spontaneously-resolving cardiomegaly in about half of the affected cases - Cardiomyopathy (rare) |
| **BIRT-HOGG-DUBÉ SYNDROME** OMIM [135150](https://omim.org/entry/135150)  **Pulmonary:**   - Numerous pleural and subpleural cysts in more than 80% of adult patients - Single or recurrent spontaneous pneumothorax, primarily occurring between 20-40 years of age - Estimated prevalence of pneumothorax about 22-38%   **Renal:**   - Kidney tumors ranging from benign oncocytomas to malignant renal cell carcinomas, chromophobe/oncocytoma hybrid renal cell cancer tumors - Tumors can be multifocal and bilateral   **Skin Lesions:**   - Fibrofolliculomas: Typically appearing between 20-40 years of age; located on the face (especially paranasal and forehead), retroauricular regions, neck, and upper trunk; present as multiple small (2-5 mm) whitish papules - Trichodiscomas - Acrochordons |
| **BLOOM SYNDROME** OMIM [210900](https://omim.org/entry/210900)  **Growth and Development:**   - Proportionate growth deficiency of prenatal onset - Average birth weight of 1757 grams - Continued growth deficiency throughout life - Average adult height of 149 cm for men and 138 cm for women   **Craniofacial Features:**   - Dolichocephaly - Narrow face - Prominent nose and ears - Malar hypoplasia - Mandibular hypoplasia   **Subcutaneous Tissue:**   - Sparse subcutaneous adipose tissue   **Skin and Dermatological Features:**   - Telangiectatic erythema appearing during the first 1-2 years of life; located on the face, particularly the cheeks, dorsum of the hands, and other sun-exposed areas - Café-au-lait macules - Hypopigmented skin lesions   **Feeding and Nutrition:**   - Slow feeding in children - Decreased appetite - Limited variety of foods consumed - Modest weight gain despite nutritional interventions - Children rarely in the normal range for growth   **Fertility and Reproductive Health:**   - Most men have azoospermia or severe oligospermia - Women are often fertile but may experience premature menopause |
| **BOHRING-OPITZ SYNDROME** OMIM [605039](https://omim.org/entry/605039)  **Growth and Development:**   - Intrauterine growth restriction - Severe neonatal feeding difficulties - Microcephaly - Trigonocephaly   **Craniofacial Features:**   - Cleft lip and/or palate - Hirsutism   Facial dysmorphism:   - Glabellar/frontal nevus flammeus - Synophrys - Proptosis - Hypertelorism - Depressed wide nasal bridge - Anteverted nares - Full cheeks - Micrognathia   **Posture and Musculoskeletal:**   - Internal rotation of the shoulders - Flexion of the elbows - Ulnar deviation of wrists and/or metacarpophalangeal joints - Truncal hypotonia - Hypertonic extremities   Joint abnormalities:   - Contractures - Congenital hip dislocations - Radial head dislocations   **Neurological and Sensory:**   - Seizures during infancy - Obstructive sleep apnea/sleep disturbances   Brain abnormalities:   - Corpus callosum defects   Eye abnormalities:   - Retinal and optic nerve abnormalities - High myopia   **Cardiovascular:**   - Bradycardia - Septal heart defects   **Gastrointestinal:**   - Cycling emesis - Gastroesophageal reflux disease |
| **CARDIOFACIOCUTANEOUS SYNDROME** OMIM [115150](https://omim.org/entry/115150)  **Prenatal:**   - Polyhydramnios   **Neonatal and Craniofacial Features:**   - Relative macrocephaly - Short webbed neck - Distinctive dysmorphic craniofacial features:   - Coarse facial appearance   - Large forehead   - Low-set ears   - Ptosis   - Downslanting eyes   - Epicanthal folds   - Short nose with depressed nasal bridge   - Prominent philtrum   - High arched palate   - Thick lower lip   **Cardiac Abnormalities:**   - Valvular pulmonary stenosis - Interauricular communication - Hypertrophic cardiomyopathy   **Gastrointestinal:**   - Feeding difficulties - Failure to thrive - Gastroesophageal reflux - Vomiting - Constipation   **Growth:**   - Growth failure leading to short stature - Sometimes due to growth hormone deficiency   **Dermatological:**   - Sparse, thin, and curly hair - Dry, hyperkeratotic and hyperelastic skin (particularly on arms, legs, and face) - General hyperpigmentation - Progressively forming nevi (moles) - Ichthyosis - Palmoplantar keratoderma - Café au lait spots - Lymphedema - Hemangiomas - Severe eczematous lesions   **Ophthalmologic:**   - Hypertelorism - Strabismus - Nystagmus - Optic nerve hypoplasia - Astigmatism - Decreased vision and visual acuity   **Ear and Hearing:**   - Recurrent otitis media   **Neurological:**   - Hypotonia - Learning difficulties - Developmental delay (motor and speech) - Seizures present in 50% of cases |
| **CBL SYNDROME** OMIM [613563](https://omim.org/entry/613563)  **Dysmorphic Facial Features:**   - High forehead - Hypertelorism - Downslanting palpebral fissures - Ptosis - Low-set ears - Prominent philtrum - Short neck with or without pterygium colli   **Neurological and Developmental:**   - Developmental delay - Hypotonia - Small head circumference   **Cardiac:**   - Congenital heart defects - Cardiomyopathy   **Ectodermal Anomalies:**   - Potential ectodermal abnormalities   **Growth:**   - Short stature   **Variability:**   - The Noonan-like phenotype can be subtle or inapparent in many individuals - In some cases, the phenotype can be severe |
| **CONSTITUTIONAL MISMATCH REPAIR DEFICIENCY SYNDROME** OMIM [619101](https://omim.org/entry/619101)  **Dermatological Features:**   - **Café-au-lait spots** - **Freckling** - **Neurofibromas**   **Gastrointestinal Features:**   - **Adenomas** - **Polyps** |
| **COSTELLO SYNDROME** OMIM [218040](https://omim.org/entry/218040)  **Birth and Early Childhood:**   - **Above-average birth weight** - **Mild hydrops** - **Severe postnatal feeding difficulties** - **Failure to thrive** - **Need for feeding tube placement**   **Developmental and Growth Features:**   - **Developmental delay** - **Short stature** - **Mild to moderate intellectual disability** - **Relative macrocephaly**   **Craniofacial and Hair Features:**   - Epicanthal folds - Full cheeks - Low set and prominent ears - Upturned nasal tip - Large mouth with prominent lips - Curly or sparse, fine hair   **Skin Features:**   - Loose, soft skin with deep palmar and plantar creases - Papillomata (on torso, extremities, perinasal, and/or perianal regions generally during childhood)   **Musculoskeletal Features:**   - Joint laxity - Ulnar deviation of wrists and fingers - Hip dysplasia - Kyphoscoliosis in older individuals - Tight Achilles tendons in later childhood (may require surgical correction) - Premature aging signs - Osteoporosis and osteopenia   **Cardiovascular Features:**   - Valvular pulmonary stenosis - Arrhythmia (ectopic or multifocal atrial tachycardia) - Hypertrophic cardiomyopathy - Rarely, aortic dilation   **Neurological Features:**   - Hydrocephalus - Seizures - Arnold-Chiari malformation type I - Syringomyelia - Tethered spinal cord   **Endocrine and Puberty Features:**   - Delayed or disordered puberty |
| **DENYS-DRASH SYNDROME** OMIM [194080](https://omim.org/entry/194080)  **Renal Features:**   - **Infantile nephrotic syndrome:** Progresses to end-stage renal disease within 1 to 15 years   **Congenital Anomalies of the Kidney and Urinary Tract:**   - Present in approximately 10% of individuals - Examples include duplex or horseshoe kidney, urogenital sinus, and vesico-ureteric reflux   **Genital Features:**   - **Ambiguous external genitalia:** Examples include hypospadias and cryptorchidism in 46,XY individuals - **Disorders of Testicular Development:** Wide range from undervirilized males to complete sex reversal in 46,XY karyotype individuals - Risk of developing gonadoblastoma in dysgenic gonads |
| **DIAMOND-BLACKFAN ANEMIA** OMIM [105650](https://omim.org/entry/105650)  **Early Life and Diagnosis:**   - Anemia discovered early, typically within the first 2 years of life - Diagnosis after 4 years of age is very unlikely   **Growth and Development:**   - Over half of patients present with short stature   Craniofacial anomalies:   - Pierre-Robin syndrome - Cleft palate - Thumb anomalies - Urogenital anomalies   **Pregnancy Considerations:**   - Pregnancies are identified as high-risk for both mother and child |
| **DICER1 SYNDROME** OMIM [601200](https://omim.org/entry/601200)  Nasal Cavity:   - Chondromesenchymal Hamartoma in childhood or early adolescence   Thyroid:   - Multinodular goiter, typically appearing between ages 10-30 years, though it can occur as early as age 5; more common in females than males; with a large proportion of female carriers developing this condition of their lifetime   Kidney:   - Cystic nephroma presenting as multicystic mass in the kidney with bimodal age distribution, occurring in children under 4 years old and in adults around the fifth decade of life; about 15% develop cystic nephroma   Ovaries:   - Ovarian-Sertoli-Leydig Cell tumor, generally benign, occasionally malignant; in young females, typically during adolescence   Pulmonary Cysts - Pleuropulmonary Blastoma (PPB) Type I:   - Purely cystic lesions typically appearing in children under one year of age - The rare form (PPB Type Ir) may be non-malignant and does not progress to the more aggressive forms (Type II and III) |
| **DYSKERATOSIS CONGENITA** OMIM [620040](https://omim.org/entry/620040)  **Triad of Classic Manifestations:**   - **Dysplastic Nails** - **Lacy Reticular Pigmentation,** primarily on the neck and upper chest - **Oral Leukoplakia**   **Bone Marrow Failure (BMF):**   - High risk of progressive BMF   **Developmental and Physical Anomalies:**   - Developmental delay - Short stature - Microcephaly   **Ocular and Dental Issues:**   - Blepharitis - Epiphora - Periodontal disease - Taurodontism - Decreased teeth/root ratio   **Gastrointestinal and Urological Issues:**   - Esophageal stenosis - Urethral stenosis   **Musculoskeletal and Dermatological Features:**   - Osteoporosis - Avascular necrosis of the femur and/or humerus - Premature hair greying/alopecia - Abnormal eyelashes   **Pulmonary and Hepatic Complications:**   - Pulmonary fibrosis - Pulmonary arteriovenous malformations - Gastrointestinal telangiectasias - Liver disease   **Progression Over Time:**   - Clinical features progress with age - The mucocutaneous triad and other features may not be present initially but develop over time |
| **FAMILIAL ADENOMATOUS POLYPOSIS** OMIM [175100](https://omim.org/entry/175100)  **Gastrointestinal Symptoms:**   - Often asymptomatic for years until adenomas are large and numerous   **Polyp Development:**   - Polyps typically appear in adolescence - Cancers generally develop about a decade after the appearance of polyps   **Extraintestinal Manifestations:**   - **Osteomas,** particularly on the skull and mandible - **Dental Abnormalities:** Supernumerary teeth, unerupted teeth, and dentigerous cysts - **Congenital Hypertrophy of the Retinal Pigment Epithelium** - **Desmoid Tumors** can occur in the abdomen, abdominal wall, or extremities   **Variants and Syndromes:**   - **Attenuated Familial Adenomatous Polyposis:**   - Fewer colorectal adenomatous polyps (usually 10 to 100)   - Later age of adenoma appearance   - Lower cancer risk - **Gardner Syndrome:**   - Association with skull and mandible osteomas, dental abnormalities, and fibromas on the scalp, shoulders, arms, and back - **Turcot Syndrome:**   - Association with medulloblastoma (a type of brain cancer) |
| **FANCONI ANEMIA** OMIM [617883](https://omim.org/entry/617883), [617243](https://omim.org/entry/617243), [617244](https://omim.org/entry/617244), [617247](https://omim.org/entry/617247), [227645](https://omim.org/entry/227645), [227646](https://omim.org/entry/227646), [227650](https://omim.org/entry/227650), [300514](https://omim.org/entry/300514), [600901](https://omim.org/entry/600901), [603467](https://omim.org/entry/603467), [609053](https://omim.org/entry/609053), [609054](https://omim.org/entry/609054), [610832](https://omim.org/entry/610832), [613390](https://omim.org/entry/613390), [613951](https://omim.org/entry/613951), [614082](https://omim.org/entry/614082), [614083](https://omim.org/entry/614083), [615272](https://omim.org/entry/615272), [616435](https://omim.org/entry/616435)  **Limb Anomalies:**   - Typically affect extremities - Can be unilateral or bilateral (usually asymmetric)   **Minor Anomalies:**   - Low birth length and weight - Microcephaly - Microphthalmia   **Skin Pigmentation Abnormalities:**   - Café-au-lait spots   **Hypoplastic Thenar Eminence:**   - Underdeveloped base of the thumb   **Ear Malformations:**   - Present in almost 20% of patients - May or may not include hearing loss   Gastrointestinal Tract Anomalies:   - Esophageal atresia, with or without tracheoesophageal fistula - Duodenal atresia - Anorectal malformations   **Congenital Malformations:**   - Can involve other organ systems - Vary within families   **Short Stature:**   - Syndromic - Often associated with endocrinopathies   **Fertility Issues:**   - Frequently impaired in males - Highly disturbed in half of females   **Bone Marrow Failure:**   - Median onset at 7 years - Develops in 90% of patients by 40 years of age - Initial signs include macrocytosis and thrombocytopenia |
| **FRASIER SYNDROME** OMIM [136680](https://omim.org/entry/136680)  **Renal Features:**   - **Nephropathy:**   - Hallmark of the disease   - Develops during childhood   - Presents as persistent proteinuria   - Progresses to steroid-resistant nephrotic syndrome   - Advances to end-stage renal disease typically in the second or third decade of life   - Focal segmental glomerulosclerosis is the most common finding on renal biopsy   **Genital and Reproductive Features:**   - **46, XY Karyotype with Female External Genitalia:**   - Complete gonadal dysgenesis   - Female external genitalia and presence of Mullerian structures   **Delayed Puberty or Primary Amenorrhea:**   - Often evaluated later for these issues - Modest breast development can occur without estrogen stimulus - Delayed puberty may be confused with effects of previous immunosuppressive therapy, renal insufficiency, or renal transplantation - **Infertility:** Complete gonadal dysgenesis results in infertility |
| **GORLIN SYNDROME or NEVOID BASAL CELL CARCINOMA SYNDROME** OMIM [109400](https://omim.org/entry/109400)  **Craniofacial and Dental Features:**   - Early onset of mandibular odontogenic keratocysts (2nd decade of life) - Macrocephaly - Frontal bossing - Coarse facial features - Facial milia - Cleft lip/palate (occasionally)   **Skin and Dermatological Features:**   - Palmar or plantar pits (asymmetrical, 2-3 mm in diameter, 1-3 mm in depth, developing in the 2nd decade)   **Skeletal Anomalies:**   - Ectopic calcification, particularly in the falx cerebri (present in more than 90% of individuals by age 20) - Fusion of vertebrae - Wedge-shaped vertebrae - Bifid or fused ribs - Hemivertebra - Kyphoscoliosis - Pectus deformity - Sprengel deformity - Syndactyly - Polydactyly   **Ocular Features:**   - Cataract - Coloboma - Microphthalmos   **Other Features:**   - Lymphomesenteric cysts   **Benign Tumors:**   - Meningioma - Papillary fibroelastoma of the heart - Ovarian fibroma (commonly bilateral and calcified) |
| **HEREDITARY LEIOMYOMATOSIS AND RENAL CELL CANCER** OMIM [150800](https://omim.org/entry/150800)  **Cutaneous Leiomyomas:**   - Onset commonly around age 25 (range 10-47 years) - Present as firm papules or nodules, skin colored to light brown - Typically localized to the trunk and extremities, but can also appear on the face - Increase in size and number with age - Sensitive to touch and/or cold temperature, often painful   **Uterine Leiomyomas:**   - Present in 77% of women - Usually appear around age 30 (range 18-52 years) - Symptoms include pelvic pain and irregular or heavy menstrual bleeding - Often diagnosed due to symptomatic presentation |
| **HYPERPARATHYROID-JAW TUMOR SYNDROME** OMIM 145001  **Parathyroid and Renal Features:**   - **Parathyroid Hyperplasia/Adenomas:**   - Development of multiple cystic parathyroid adenomas.   - Recurrent adenomas several years post-resection.   **Hypercalcemia:**   - Persistent elevated calcium levels post-adenoma resection. - Hypercalciuria   **End-Stage Renal Disease:**   - Associated with severe nephropathy.   **Renal Cysts and Hamartomas:**   - Prominent in some families, possibly representing a new phenotypic variant   **Skeletal and Jaw Features:**   - **Ossifying Jaw Fibromas:**   - Maxillary and mandibular tumors, histologically distinct fibroosseous lesions without giant cells.   - Different from "brown tumors" typically associated with hyperparathyroidism.   **Genital and Reproductive Features:**   - Urolithiasis (kidney stones) - Multiple small adenomyomatous polyps observed in some female patients.   **Disorders of Sexual Development:**   - Instances of male predominance |
| **JUVENILE POLYPOSIS SYNDROME** OMIM [174900](https://omim.org/entry/174900)  **Polyp Development:**   - Can develop at any age from infancy through adulthood - Most affected individuals present with polyps by adolescence or early adulthood   **Diagnostic Criteria:**   - Presence of more than five juvenile polyps in the colon and/or rectum - Presence of juvenile polyposis throughout the digestive tract, including the stomach - Any number of juvenile polyps in association with a family history   **Other Associated Signs:**   - Growth delay - Edema |
| **MUIR-TORRE SYNDROME** OMIM [158320](https://omim.org/entry/158320)  **Dermatological Features:**   - **Sebaceous Skin Tumors:**   - Sebaceous adenomas   - Sebaceous epitheliomas   - Basal cell epitheliomas with sebaceous differentiation - **Keratoacanthomas** |
| **MULIBREY NANISM** OMIM [253250](https://omim.org/entry/253250)  **Growth and Development:**   - Pre- and postnatal growth restriction - Relative macrocephaly - Psychomotor development is mainly normal   **Craniofacial Features:**   - Scaphocephaly - Facial triangularity - Broad forehead - Low nasal bridge   **Body and Extremities:**   - Thin extremities - Feeding difficulties in infants - Children are prone to respiratory problems   **Ocular Features:**   - Yellowish dots in the retinal mid-peripheral region   **Voice and Skin:**   - High-pitched voice - Cutaneous naevi flammei   **Radiological Findings:**   - Slender long bones with thick cortex and narrow medullary channel - Fibrous dysplasia - J-shaped sella turcica - Small thoracic cage   **Cardiovascular:**   - Restrictive perimyocardial heart disease   **Liver and Metabolic:**   - Hepatomegaly - Fatty liver - Insulin resistance, with over 90% of adults showing abnormally high fasting insulin levels, resulting in type 2 diabetes in half of the patients   **Reproductive Health:**   - Primary hypogonadism (both male and female) - Infertility |
| **MULTIPLE ENDOCRINE NEOPLASIA TYPE 1** OMIM [131100](https://omim.org/entry/131100)  **Tumor Development:**   - Tumors can develop at any age, with 95% of patients developing clinical symptoms by the 5th decade.   **Parathyroid Tumors:**   - Most common, occurring in 95% of patients - Primary Hyperparathyroidism (PHPT) is the most common initial manifestation, with a mean age of onset in the third decade of life. - PHPT can present as normocalcemic (asymptomatic) or with hypercalcemia.   **Pancreatic Islet Tumors:**   - Occur in 40% of patients - Types of Pancreatic Neuroendocrine Tumors:   - Gastrinoma (50%): Can cause peptic ulcers and Zollinger-Ellison syndrome   - Insulinoma (33%)   - Glucagonoma (5%)   - VIP-oma   - Pancreatic Polypeptide-oma   - Non-functioning tumors   **Anterior Pituitary Tumors:**   - Occur in 30% of patients - Types of Pituitary Tumors:   - Prolactinoma (66%)   - Somatotrophinoma (25%)   - ACTHoma (5%)   - Non-functioning adenomas (5%) |
| **MULTIPLE ENDOCRINE NEOPLASIA TYPE 2A** OMIM [171400](https://omim.org/entry/171400)  **Primary Conditions:**   - **Pheochromocytoma** - **Primary Hyperparathyroidism**   **Age of Onset:**   - Typically before 35 years of age - Later onset compared to MEN2B   **Gastrointestinal Symptoms:**   - Diarrhea is the most frequent systemic symptom   **Associated Conditions:**   - **Hirschsprung Disease** - **Cutaneous Lichen Amyloidosis** - **Excessive Production of Adrenocorticotropic Hormone** |
| **MULTIPLE ENDOCRINE NEOPLASIA TYPE 2B** OMIM [162300](https://omim.org/entry/162300)  **Primary Conditions:**   - **Pheochromocytoma**   **Craniofacial and Oral Features:**   - Typical facies with:   - Mucosal neuromas of the lips and tongue   - Bumpy lips   **Ophthalmologic Abnormalities:**   - Alacrima in infancy - Thickened and everted eyelids - Mild ptosis - Prominent corneal nerves   **Skeletal Anomalies:**   - Marfanoid habitus - Narrow, long facies - Pes cavus - Pectus excavatum - High-arched palate - Scoliosis - Hyperextensible joints - Slipped capital femoral epiphyses   **Gastrointestinal Features:**   - Generalized ganglioneuromatosis throughout the aerodigestive tract - Chronic constipation - Abdominal distension - Diarrhea - Megacolon at birth |
| **NEUROFIBROMATOSIS TYPE 1** OMIM [162200](https://omim.org/entry/162200)  **Dermatological Features:**   - Multiple café-au-lait macules present in almost all patients:   - Some present at birth   - Most develop before the first year of life - Intertriginous freckling, starting at 5 years of age - Multiple cutaneous and subcutaneous neurofibromas develop in adults and continue to increase in number and size with age   Plexiform neurofibromas:   - Usually present at birth   **Ocular Manifestations:**   - Optic pathway gliomas: Develop usually before age 6 years - Iris hamartomas   **Skeletal and Growth Abnormalities:**   - Osteopenia and osteoporosis - Short stature - Macrocephaly - Scoliosis - Skeletal dysplasia (sphenoid wing, vertebral anomalies) - Pseudoarthrosis   **Cardiovascular and Other Systemic Features:**   - Hypertension - Vasculopathy - Occasionally, seizures or hydrocephalus   **Cognitive and Developmental Features:**   - Intellectual development is usually not severely affected - Cognitive deficits and learning difficulties are frequent (50%-75%) |
| **NEUROFIBROMATOSIS TYPE 2** OMIM [101000](https://omim.org/entry/101000)  **Neurological and Auditory Features:**   - **Schwannomas:** Typically affect both vestibular nerves, leading to hearing loss and deafness   **Other Tumors:**   - Schwannomas of other cranial, spinal, and peripheral nerves - Meningiomas, both intracranial (including optic nerve meningiomas) and intraspinal   **Ophthalmic Features:**   - Reduced visual acuity - Cataracts   **Dermatological Features:**   - About 70% of NF2 patients have skin tumors:   - Intracutaneous plaque-like lesions   - Deep-seated subcutaneous nodular tumors |
| **NIJMEGEN BREAKAGE SYNDROME** OMIM [251260](https://omim.org/entry/251260)  **Growth and Development:**   - Microcephaly present at birth and progressing with age - Mild growth retardation - Premature ovarian insufficiency in females   **Craniofacial Features:**   - Dysmorphic facial features:   - Prominent midface   - Sloping forehead   - Receding mandible   **Congenital Anomalies:**   - **Central Nervous System:**   - Hydrocephaly   - Schizencephaly   - Arachnoid cysts - **Respiratory Tract:**   - Cleft lip/palate   - Choanal atresia - **Urogenital System:**   - Horseshoe kidney   - Ectopic/dystopic kidneys   - Hypospadias   - Cryptorchidism   - Ovary hypoplasia - **Skeletal Anomalies:**   - Pre- and postaxial polydactyly   - Hypoplastic or duplicated thumb   - Clinodactyly of 5th fingers   **Dermatological Features:**   - Café au lait spots - Vitiligo spots - Multiple pigmented nevi in some patients   **Immune System:**   - Immune deficiency with recurrent respiratory tract infections   **Cognitive Development:**   - Close to average (normal/borderline) in infancy and preschool age - Intellectual skills gradually decline with age (from mild to moderate) |
| **NOONAN SYNDROME** OMIM [618624](https://omim.org/entry/618624)  **Neonatal and Infancy Features:**   - Feeding difficulties - Failure to thrive   Characteristic facial features becoming more obvious in infancy:   - High broad forehead - Hypertelorism - Palpebral ptosis - Downward slanting palpebral fissures - Low-set, thick, posteriorly rotated ears - Deep philtrum - Micrognathia - Curly hair - Short neck, sometimes with pterygium colli - With age, face becomes triangular with marked skinfolds   **Cardiovascular Features:**   - Pulmonary valve stenosis (50-60%) with pulmonic valve dysplasia - Various cardiac malformations (atrial septal defects, ventricular septal defects) - Hypertrophic cardiomyopathy, common in antenatal period (20%) - Dilation of coronary arteries - Moyamoya disease development with aging   **Growth and Development:**   - Growth delay affects 50%, uncommonly associated with growth hormone deficiency - Difficulty in weight gain; many patients remain lean - Delayed motor development - Delayed puberty - Short stature present in 50%   **Orthopedic Manifestations:**   - Sternal deformity - Talipes equinovarus - Progressive scoliosis (onset in adolescence)   **Dermatological Features:**   - Dry skin - Hyperkeratosis on hands and feet - Curly hair, which may be thick or sparse   **Lymphedema:**   - Peripheral lymphedema, possibly progressive and extensive   **Ocular and Dental Features:**   - Ocular anomalies (strabismus, refractive errors) - Dental crowding   **Hearing and Speech:**   - Hearing loss in 10% - Delayed speech - Learning difficulties in 30-40% - Intellectual disability (often mild) in 10-20%   **Neurological and Behavioral Features:**   - Dyspraxia - Attention deficit disorder - Agitation, mood disorders, and emotional disturbances - Difficulties in identifying and expressing emotions, impacting social interactions   **Reproductive Health:**   - Unilateral or bilateral cryptorchidism in two-thirds of boys - Hypofertility may affect males, but not females   **Thyroid and Coagulation:**   - Thyroid dysfunction may occur - Coagulation defects are frequent but rarely clinically significant |
| **PERLMAN SYNDROME** OMIM [267000](https://omim.org/entry/267000)  **Facial Dysmorphism:**   - Upsweeping anterior scalp hair - Depressed nasal bridge - Hypotonic appearance with an open mouth - Prominent everted upper lip - Mild micrognathia   **Neurological Features:**   - Agenesis of the corpus callosum - Choroid plexus hemangiomas   **Craniofacial Features:**   - Cleft palate   **Cardiovascular Anomalies:**   - Dextroposition of the heart - Interrupted aortic arch   **Thoracic and Abdominal Features:**   - Diaphragmatic hernia - Visceromegaly: Nephromegaly, hepatomegaly, cardiomegaly, thymus hyperplasia - Hepatic fibrosis with porto-portal bridging - Abdominal muscular hypoplasia - Distal ileal atresia   **Genitourinary Anomalies:**   - Cryptorchidism   **Metabolic Features:**   - Hyperinsulinism, an important feature and potentially preventable cause of death |
| **PEUTZ-JEGHERS SYNDROME** OMIM [175200](https://omim.org/entry/175200)  **Gastrointestinal Features:**   - **Hamartomatous Polyps:**   - Generally occur in childhood and early adulthood, often with onset during the first 10 years of life.   - Can occur at any site in the GI tract, most frequently in the small intestine.   - Other potential sites include the stomach, large intestine, nares, and rarely the renal pelvis, urinary bladder, and lungs.   - Presence of adenomas and hyperplastic polyps.   **Mucocutaneous Pigmentation:**   - Development of dark blue to dark brown macules around the mouth, eyes, nares, perianal area, and buccal mucosa during infancy or childhood. - Hyperpigmentation may also be found on the fingers and toes. - Lesions may fade in adolescence and adulthood but tend to persist in the buccal mucosa.   **Other Features:**   - Female patients may also develop typically benign bilateral multifocal sex cord tumors with annular tubules (SCTAT). |
| **PTEN Hamartoma Tumor Syndrome**OMIM [158350](https://omim.org/entry/175200)  **General Features:**   - **Macrocephaly** - **Benign thyroid pathology, e**specially hashimoto thyroiditis   **Dermatological and Gastrointestinal Features:**   - **Mucocutaneous hamartomas** - **Colonic polyps**   **Vascular Features:**   - **Vascular malformations**   ***Cowden Syndrome:***   - Mucocutaneous lesions, macrocephaly, breast and thyroid abnormalities   ***Proteus-like Syndrome:***   - Asymmetrical overgrowth, skin lesions, and vascular malformations   ***Lhermitte-Duclos Disease:***   - Dysplastic gangliocytoma of the cerebellum   ***Segmental Overgrowth-Lipomatosis-Arteriovenous Malformation-Epidermal Nevus (SOLAMEN) Syndrome:***   - Overgrowth, lipomas, arteriovenous malformations, and epidermal nevi |
| **ROTHMUND-THOMSON SYNDROME** OMIM [268400](https://omim.org/entry/268400)  **Dermatological Features:**   - **Erythema:** Develops on cheeks at 3-6 months of age, spreads to extremities and buttocks, sparing trunk and abdomen - **Cutaneous Atrophy:** Reticulated areas of hypo- and hyperpigmentation, persistent telangiectasias - **Other Skin Manifestations:**   - Dental anomalies   - Nail dystrophy   - Palmo-plantar hyperkeratotic lesions   ***RTS Type 1:***   - Poikiloderma - Ectodermal dysplasia (affecting hair, teeth, nails, and sweat glands) - Juvenile cataracts   ***RTS Type 2:***   - Poikiloderma - Congenital bone defects (frontal bossing, saddle nose, radial ray defects like thumb hypo- or aplasia or radial aplasia)   **Extracutaneous Manifestations:**   - **Gastrointestinal:**   - Chronic emesis   - Diarrhea - **Respiratory Issues** - **Hematological:** Anemia, neutropenia, myelodysplasia - **Endocrine:** Hypogonadism - **Skeletal:** Osteopenia |
| **RUBINSTEIN-TAYBI SYNDROME** OMIM [180849](https://omim.org/entry/180849)  **Facial Features:**   - Highly arched eyebrows - Long eyelashes - Downslanting palpebral fissures - Convex nasal ridge - Low hanging columella - Highly arched palate - Micrognathia - Talon cusps on permanent incisors - Unusual smile with almost complete closure of the eyes   **Eye Anomalies:**   - Nasolacrimal duct obstruction - Congenital glaucoma - Refractive errors   **Cardiovascular Features:**   - Ventricular septal defect - Atrial septal defect - Patent ductus arteriosus   **Musculoskeletal Features:**   - Joint hypermobility - Skin anomalies, particularly keloid formation   **Gastrointestinal and Respiratory Features:**   - Feeding difficulties in the first year - Frequent respiratory tract infections in infancy and childhood - Chronic constipation - Tendency to become overweight during late childhood or early puberty   **Behavioral and Psychological Features:**   - Marked ability to establish excellent social contacts in childhood - Sudden mood changes and obsessive-compulsive behavior becoming more frequent in adulthood |
| **SCHINZEL-GIEDION SYNDROME** OMIM [269150](https://omim.org/entry/269150)  **Facial Dysmorphism:**   - Prominent forehead - Midface retraction resembling a broadened "figure-of-eight" - Short upturned nose   **Visceral Abnormalities:**   - Hydronephrosis (91% of cases) - Cardiac abnormalities (43% of cases):   - Septal defects   - Valvular dysplasias   - Hypoplastic ventricles   - Patent ductus arteriosus   **Genitourinary Abnormalities (76% of cases):**   - Cryptorchidism - Micropenis - Hypospadias - Hypoplastic uterus - Hypoplastic labia minora and majora - Deep labial sulcus - Anteriorly displaced anus   **Limb Malformations:**   - Short limbs - Valgus or varus foot deformity - Mesomelic brachymelia - Hypoplastic and hyperconvex nails - Single palmar creases on the hands   **Neurological and Developmental Features:**   - Hypotonia - Respiratory failure - Severe developmental delay - Seizures (often refractory) - Visual and hearing impairment   **Additional Features:**   - Hypertrichosis - Higher prevalence of neuroepithelial tumors (17%) |
| **SHWACHMAN-DIAMOND SYNDROME** OMIM [260400](https://omim.org/entry/260400)  **Hematological Features:**   - Intermittent and moderate neutropenia (most common anomaly) - Recurrent infections due to neutropenia - Mild anemia - Thrombocytopenia   **Pancreatic and Gastrointestinal Features:**   - Exocrine pancreatic insufficiency - Failure to thrive - Growth retardation - Chronic steatorrhea   **Bone and Skeletal Features:**   - Delayed bone age and maturation - Metaphyseal dysplasia - Short stature - Pectus carinatum - Generalized osteopenia   **Dermatological and Dental Features:**   - Eczema or ichthyosis - Dental anomalies   **Neurological and Developmental Features:**   - Psychomotor retardation - Intellectual disability in 50% of patients, causing learning difficulties   **Neonatal Period:**   - Generally no symptoms observed - Some cases reported with:   - Pancytopenia   - Respiratory distress   - Severe spondylometaphyseal dysplasia   **Complications:**   - Hematologic complications may include bone marrow aplasia |
| **SIMPSON-GOLABI-BEHMEL SYNDROME** OMIM [312870](https://omim.org/entry/312870)  **Growth and Development:**   - Pre- and postnatal overgrowth with macrosomia - Macrocephaly   **Craniofacial Features:**   - Coarse facial features - Macroglossia - Hypertelorism - Dental malocclusion - Palatal abnormalities (e.g., cleft palate)   **Skin and External Features:**   - Supernumerary nipples (extra nipples)   **Cardiovascular Features:**   - Congenital heart defects - Arrhythmias   **Skeletal Features:**   - Vertebral segmental defects - Polydactyly or brachydactyly of the hands - Cutaneous syndactyly - Nail hypoplasia   **Abdominal Features:**   - Abdominal visceromegaly: Renal dysplasia/nephromegaly, splenomegaly, hepatomegaly - Diaphragmatic hernia - Diastasis recti/umbilical hernia   **Genital Features:**   - Cryptorchidism - Hypospadias   **Neurological and Developmental Features:**   - Variable degrees of intellectual disability - Motor delay - Speech delay |
| **SECKEL SYNDROME OR BEARD-HEADED DWARFISM** OMIM [210600](https://omim.org/entry/210600)  **Growth and Development:**   - Proportionate dwarfism of prenatal onset - Severe microcephaly   **Craniofacial Features:**   - Bird-headed like appearance - Distinctive facial features associated with microcephaly   **Neurological and Cognitive Features:**   - Mental retardation   **Hematological Features:**   - Hematological abnormalities with chromosome breakage found in 15 to 25% of patients |
| **SOTOS SYNDROME** OMIM [617169](https://omim.org/entry/617169)  **Growth and Development:**   - Excessive growth evident throughout life, especially in childhood - Manifestation of excessive growth can begin in fetal life - Final adult height is typically beyond or in the upper part of normal ranges - Striking macrocephaly, often disproportionate to height   **Craniofacial Features:**   - Long narrow face - Flushed cheeks - Prominent forehead with frontotemporal hair scarcity - Down-slanting palpebral fissures - Hypertelorism - High arched palate - Pointed chin   **Neurological and Cognitive Features:**   - Mild to severe intellectual disability - Wide spectrum of behavioral disorders - Developmental milestones commonly delayed - Hypotonia in the neonatal period - Seizures and electroencephalogram abnormalities   **Skeletal and Dental Features:**   - Advanced bone age - Scoliosis - Prognathia - Premature dental eruption - Large hands and feet   **Feeding and Growth:**   - Poor feeding in the neonatal period   **Hearing and Cardiac Features:**   - Less common conductive hearing loss - Possible cardiac anomalies   **Genitourinary Anomalies:**   - Less common genitourinary anomalies |
| **TUBEROUS SCLEROSIS COMPLEX** OMIM [191100](https://omim.org/entry/191100)  **Dermatological Features:**   - **Hypomelanotic macules:** Appear in the first years of life - **Facial angiofibromas:** Develop by 3-4 years - **Ungual fibromas** - **Cephalic and lumbar fibrous plaques:** Develop in childhood to early adolescence - **"Confetti" skin lesions:** Small, light-colored spots on the skin   **Neurological Features:**   - **Cortical dysplasias (tubers)** - **Subependymal nodules** - **Subependymal giant cell astrocytoma (SEGA):** Affects 10-20% of patients, mostly children and young adults - **Early-onset epilepsy:** Present in 85% of patients, including focal seizures and infantile spasms - **TSC-associated neuropsychiatric disorders:**   - Intellectual disability   - Attention-deficit/hyperactivity disorder   - Autism spectrum disorders   - Psychiatric disorders   - Neuropsychological deficits   - School and occupational difficulties   **Renal Features:**   - **Renal Angiomyolipomas:** Develop during childhood, risk of growth in adolescence and adulthood   **Pulmonary Features:**   - **Lymphangioleiomyomatosis** - **Multifocal Micronodular Pneumocyte Hyperplasia** - **Pulmonary Cysts:** Develop during adulthood - **Pulmonary Symptoms:** Dyspnea, pneumothorax, or chylothorax   **Cardiac Features:**   - **Cardiac Rhabdomyomas:** Appear during the fetal period, rarely symptomatic, tend to decrease in size in early childhood   **Additional Features:**   - **Retinal Hamartomas** - **Liver Hamartomas** - **Dental Enamel Pitting** - **Intraoral Fibromas** - **Skeletal Dysplasia** - **Neuroendocrine Tumors:** Rarely present |
| **VON HIPPEL-LINDAU SYNDROME** OMIM [193300](https://omim.org/entry/193300)  **Ocular Features:**   - **Retinal Hemangioblastomas:** Most common presenting feature; multiple and bilateral in about 50% of cases   **Central Nervous System (CNS) Features:**   - **CNS Hemangioblastomas:** Presenting feature in about 40% of cases; occur in 60-80% of patients; most often located in the cerebellum, but also in the brainstem and spinal cord   **Renal Features:**   - **Multiple renal cysts:** Very common   **Endocrine Features:**   - **Pheochromocytomas**   **Genitourinary Features:**   - **Epididymal cysts and cystadenomas:** Occur in 60% of male patients   **Gastrointestinal Features:**   - **Multiple pancreatic cysts:** Present in most patients - **Non-secretory pancreatic islet cell tumors:** Occur in 10-15% of patients   **Auditory Features:**   - **Endolymphatic sac tumors (ELST):** Found in up to 10% of patients; may cause hearing loss   **Head and Neck Features:**   - **Paragangliomas:** Rare, occurring in 0.5% of patients |
| **WAGR SYNDROME** OMIM [194072](https://omim.org/entry/194072)  **Ocular Features:**   - **Congenital Aniridia:**   - Variable severity, almost always present at birth   - Associated with other eye abnormalities such as:     - Cataract     - Glaucoma     - Limbal insufficiency     - Optic nerve hypoplasia     - Corneal opacification/vascularization   - Leading to visual impairment   **Renal Features:**   - **End-stage renal disease:** Especially at risk when presenting with Wilms tumor (45-60% of patients)   **Genitourinary and Neurological Features:**   - Genitourinary anomalies - Neurological abnormalities - Variable intellectual disability or behavioral abnormalities   **Obesity:**   - Many patients develop obesity - **WAGRO Syndrome:** WAGR syndrome with childhood-onset obesity |
| **WEAVER SYNDROME** OMIM [277590](https://omim.org/entry/277590)  **Growth and Development:**   - **Tall Stature:** ≥ two standard deviations above the mean; found in 90% of variant-positive individuals - **High Birth Weight and Length:** Most affected patients have higher than normal measurements at birth   **Craniofacial Features:**   - **Subtle but characteristic facial appearance:** Most easily recognized in early childhood - **Ocular hypertelorism** - **Large fleshy ears** - **Retrognathia**   **Neurological and Cognitive Features:**   - **Intellectual Disability:** Mild in about 80% of patients; moderate in a smaller number, with a greater impact on autonomy; severe deficits are rare   **Skeletal and Musculoskeletal Features:**   - **Macrocephaly** - **Joint laxity** - **Scoliosis:** Ranges from mild to severe - **Pectus excavatum** - **Hypotonia and hypertonia** - **Poor coordination** - **Soft skin**   **Hand and Foot Features:**   - **Clinodactyly** - **Camptodactyly**   **Other Physical Features:**   - **Umbilical hernia** - **Hoarse low cry in infancy** |
| **WERNER SYNDROME** OMIM [277700](https://omim.org/entry/277700)  **Growth and Development:**   - Normal at birth and during childhood - Absence of a pubertal growth spurt - Short stature   **Onset and major symptoms (Ages 20-30):**   - **Early onset bilateral cataracts** - **Thinning and graying of hair**   **Skin Changes:**   - Ankle ulceration - Hyperkeratosis - Tight skin - Age spots - "Bird-like" facies - Subcutaneous atrophy   **Age-Related Disorders:**   - Osteoporosis - Diabetes mellitus - Mesenchymal neoplasms - Atherosclerosis   **Additional Features:**   - Voice changes - Flat feet |
| **XERODERMA PIGMENTOSUM** OMIM [278700](https://omim.org/entry/278700)  **Variable Severity and Age of Onset:**   - Highly variable clinical manifestations - Age of onset depends on sunlight exposure and complementation group   **Sun Sensitivity:**   - Approximately 50% of affected individuals have acute sun sensitivity from the first few months of life - Others gradually develop marked freckling at sun-exposed sites without a sunburn reaction   **Skin Features:**   - Dry skin - Hypo- or hyperpigmented lesions   **Ocular Features:**   - **Keratitis** - **Photophobia**   **Ocular Tumors:**   - Ocular melanoma   **Neurological Abnormalities:**   - Reported in about 30% of cases - **Acquired microcephaly** - **Diminished or absent deep tendon stretch reflexes** - **Progressive sensorineural hearing loss** - **Spasticity** - **Ataxia** - **Seizures** - **Progressive cognitive impairment** |

**Supplemental Table 3.** Craniofacial dysmorphic features and associated cancer predisposition syndromes

| **Craniofacial Feature** | **Cancer Predisposition Syndrome** |
| --- | --- |
| Anterior earlobe crease | Beckwith-Wiedemann Syndrome |
| Appearance, beard-headed | Seckel Syndrome |
| Cheeks, flushed | Sotos Syndrome |
| Cheeks, full | Bohring-Opitz Syndrome, Costello Syndrome |
| Chin, pointed | Alagille Syndrome, Sotos Syndrome |
| Coarse facial appearance | Cardiofaciocutaneous Syndrome, Simpson-Golabi-Behmel Syndrome |
| Columella, low hanging | Rubinstein-Taybi Syndrome |
| Choanal atresia | Nijmegen-Breakage Syndrome |
| Dolichocephaly | Bloom Syndrome |
| Ear malformation | Fanconi Anemia |
| Ears, large fleshy | Weaver Syndrome |
| Ears, low-set | Cardiofaciocutaneous Syndrome, CBL Syndrome, Costello Syndrome, Noonan Syndrome |
| Ears, posteriorly rotated | Noonan Syndrome |
| Ears, prominent | Bloom Syndrome |
| Ears, thick | Noonan Syndrome |
| Epicanthal folds | Cardiofaciocutaneous Syndrome, Costello Syndrome |
| Eyebrows, highly arched | Rubinstein-Taybi Syndrome |
| Eyelashes, long | Rubinstein-Taybi Syndrome |
| Eyelids, thickened/everted | MEN2B |
| Eyes, deep-set | Alagille Syndrome |
| Eyes, downslanting | Cardiofaciocutaneous Syndrome |
| Face, narrow | Bloom Syndrome |
| Facial triangularity | Mulibrey Nanism, Noonan Syndrome |
| Facies, bird-like | Werner Syndrome |
| Facies, narrow long | MEN2B, Sotos Syndrome |
| Forehead, broad | Mulibrey Nanism, Noonan Syndrome |
| Forehead, high | CBL Syndrome, Noonan Syndrome |
| Forehead, large | Cardiofaciocutaneous Syndrome |
| Forehead, prominent | Alagille Syndrome, Schinzel-Giedion Syndrome, Sotos Syndrome |
| Forehead, sloping | Nijmegen-Breakage Syndrome |
| Frontal bossing | Gorlin Syndrome |
|  |  |
| Hypertelorism | Alagille Syndrome, Bohring-Opitz Syndrome, Cardiofaciocutaneous Syndrome, CBL Syndrome, Noonan Syndrome, Simpson-Golabi-Behmel Syndrome, Sotos Syndrome, Weaver Syndrome |
| Lip, cleft | Bohring-Opitz Syndrome, Diamond-Blackfan Anemia, Gorlin Syndrome, Nijmegen-Breakage Syndrome |
| Lip, thick lower | Cardiofaciocutaneous Syndrome |
| Lips, bumpy | MEN2B |
| Lips, prominent | Costello Syndrome |
| Macrocephaly | Bannayan-Riley-Ruvalcaba Syndrome, Cardiofaciocutaneous Syndrome, Costello Syndrome, Gorlin Syndrome, Mulibrey Nanism, NF1, PTEN Hamartoma Tumor Syndrome, Simpson-Golabi-Behmel Syndrome, Seckel Syndrome, Weaver Syndrome |
| Macroglossia | Beckwith-Wiedemann Syndrome, Simpson-Golabi-Behmel Syndrome |
| Malar hypoplasia | Bloom Syndrome |
| Mandibular hypoplasia | Bloom Syndrome |
| Mandible, receding | Nijmegen-Breakage Syndrome |
| Microcephaly | Bohring-Opitz Syndrome, Dyskeratosis Congenita, Fanconi Anemia, Nijmegen-Breakage Syndrome, Seckel Syndrome, Xeroderma Pigmentosum |
| Micrognathia | Bohring-Opitz Syndrome, Noonan Syndrome, Perlman Syndrome, Rubinstein-Taybi Syndrome |
| Midface, prominent | Nijmegen-Breakage Syndrome |
| Midface, retraction | Schinzel-Giedion Syndrome |
| Mouth, large | Costello Syndrome |
| Nares, anteverted | Bohring-Opitz Syndrome, Costello Syndrome |
| Nasal root, flat | Alagille Syndrome |
| Nasal bridge, low | Mulibrey Nanism |
| Nasal bridge, depressed (wide) | Bohring-Opitz Syndrome, Cardiofaciocutaneous Syndrome, Perlman Syndrome |
| Nasal ridge, convex | Rubinstein-Taybi Syndrome |
| Neck, short (webbed) | Cardiofaciocutaneous Syndrome, CBL Syndrome, Noonan Syndrome, Schinzel-Giedion Syndrome |
| Nose, prominent | Bloom Syndrome |
| Nose, upturned | Schinzel-Giedion Syndrome |
| Palate, cleft | Bohring-Opitz Syndrome, Diamond-Blackfan Anemia, Gorlin Syndrome, Nijmegen-Breakage Syndrome, Perlman Syndrome, Simpson-Golabi-Behmel Syndrome |
| Palate, high arched | Cardiofaciocutaneous Syndrome, MEN2B, Rubinstein-Taybi Syndrome, Sotos Syndrome |
| Palpebral fissures, upslanting | Alagille Syndrome |
| Palpebral fissure, downslanting | CBL Syndrome, Noonan Syndrome, Rubinstein-Taybi Syndrome, Sotos Syndrome |
| Philtrum, deep | Noonan Syndrome |
| Philtrum, prominent | Cardiofaciocutaneous Syndrome, CBL Syndrome |
| Pierre-Robin Syndrome | Diamond-Blackfan Anemia |
| Posterior helical pit | Beckwith-Wiedemann Syndrome |
| Prognathia | Sotos Syndrome |
| Proptosis | Bohring-Opitz Syndrome |
| Pterygium colli | Noonan Syndrome |
| Ptosis | Cardiofaciocutaneous Syndrome, CBL Syndrome, MEN2B, Noonan Syndrome |
| Retrognathia | Weaver Syndrome |
| Scalp hair, upsweeping anterior | Perlman Syndrome |
| Scaphocephaly | Mulibrey Nanism |
| Smile, unusual | Rubinstein-Taybi Syndrome |
| Synophrys | Bohring-Opitz Syndrome |
| Talon cusp | Rubinstein-Taybi Syndrome |
| Trigonocephaly | Bohring-Opitz Syndrome |
